# One-stop strabismus digital diagnosis via AI-integrated skin-like and wearable “Eyelectronics”

**DOI:** 10.1126/sciadv.aeb7242

**Published:** 2026-01-28

**Authors:** Yong Yang, Xin Liu, Jiankai Tang, Hengyi Guo, Jinsong Zhang, Yuntao Wang, Yonghong Jiao, Yihao Chen, Xue Feng

**Affiliations:** ^1^ State Key Laboratory of Flexible Electronics Technology, Tsinghua University, Beijing 100084, China.; ^2^ AML, Department of Engineering Mechanics, Tsinghua University, Beijing 100084, China.; ^3^ Institute of Flexible Electronics Technology of THU, Zhejiang, Jiaxing 314000, Zhejiang, China.; ^4^ Department of Computer Science and Technology, Tsinghua University, Beijing 100084, China.; ^5^ School of Computer Science and Technology, Qinghai University, Xining 810016, Qinghai, China.; ^6^ Beijing Tongren Eye Center, Beijing Tongren Hospital, Capital Medical University, Beijing 100730, China.; ^7^ Beijing Ophthalmology and Visual Science Key Lab, Beijing 100069, China.

## Abstract

Strabismus, affecting ~4% of children, impairs vision and psychosocial health. However, clinical diagnosis requires multiple instruments and stepwise examinations of ocular alignment, extraocular muscle function, and deviation angle. It is limited by low diagnostic objectivity, poor pediatric compliance, and high cost. Here, we propose a strategy for one-stop strabismus digital diagnosis via artificial intelligence (AI)–integrated, skin-like, and wearable “Eyelectronics.” The ultralightweight, imperceptible eye-wearable system features an ultrathin (~60 micrometers in thickness), breathable, and multidirectional (0°/45°/90°) strain-sensing array conformally adapted to the sensitive eyelid. It enables wireless, mild-restricted measurement of eyelid deformation during eye movements. Through biomechanical modeling validated by ocular magnetic resonance imaging simulations, we establish a prior correlation between eyelid deformation and eye movements. The Eyelectronics, powered by our physiology knowledge-driven end-to-end AI algorithm, achieves simultaneous measurement of strabismus angle and identification of paretic muscle. It delivers a 96.6% four-direction classification accuracy and a 1.2° measurement accuracy in ocular motility examinations. Clinical benchmarking against the clinical standard (Hess screen test) confirmed diagnostic agreement (intraclass correlation = 0.978). This system bridges quantitative biomechanical sensing with digital diagnosis, promoting a paradigm for future strabismus treatment.

## Teaser

Skin-like wearable electronics with integrated AI provide a one-stop, objective, and accurate digital diagnosis for strabismus.

## INTRODUCTION

Strabismus, affecting 2 to 4% of children globally, causes ocular misalignment and functional impairments due to extraocular muscle (EOM) dysfunction, neurological issues, or trauma (*1*–*3*). Surgical correction adjusting EOM tension remains primary for moderate/severe cases, yet 20 to 50% require reoperations due to inaccurate strabismus angle measurement (*4*). Therefore, accurate strabismus measurement and function evaluation of EOMs are paramount to the diagnosis and subsequent surgery. Clinically, strabismus measurement is usually achieved through the prism-cover test, whereas the Hirschberg and Krimsky tests serve as supplementary methods. The prism-cover test measures the strabismus angle in a single direction, whereas strabismus angles often result from the combined influence of multiple EOMs. Consequently, the prism-cover test has inherent limitations. Function evaluation of EOMs is aided by mapping of strabismus angles in all nine gaze directions. The Hess screen test is often used as the clinical standard to localize the EOM lesions using red-green glasses to break binocular fusion (*5*). It provides deviation angles across multiple gaze positions, offering a more comprehensive view of ocular misalignment. The Lees and Lancaster screen tests are further modifications of the Hess screen test. The Harms test is a similar but more cumbersome test requiring rotating the head into nine positions. Even with adequate patient cooperation, these tests have inherent limitations in accuracy due to their reliance on subjective patient responses for localizing the light position on the screen. Now, there is a lack of a one-stop solution for simultaneous accurate strabismus measurement and EOM function evaluation in clinical practice. Moreover, all of them mainly rely on the doctor’s experience and subjective judgment (*5*–*7*). It is also difficult for children to cooperate due to the discomfort caused by the rigid device and the complicated testing procedures. Accurate strabismus diagnosis thus requires the coordination of multiple factors, including doctor’s judgment, patient’s subjective participation, and cumbersome instruments. These limitations may lead to large errors and poor repeatability, highlighting the urgent need for a one-stop strabismus digital diagnosis.

To enhance reproducibility and objectivity, researchers have explored objective strabismus measurement methods, mainly including eye tracking methods to simulate clinical methods based on vision and electrooculography (EOG) (*8*, *9*). Weber *et al.* designed novel simplified video goggles tailored for noninvasive assessment of strabismus (*10*). However, its reliance on costly and bulky components, including high-resolution cameras and mounting units, presents hurdles for miniaturization (*11*). Camera-based systems also entail frequent calibration and suffer from ambient light interference. Moreover, shifts in the relative positions of the camera and the head compromise the accuracy of eye tracking (*12*). Alternatively, EOG has emerged as a promising technique, yet it contends with signal-to-noise limitations and instability (*13*–*15*). To improve the measurement accuracy, Mishra *et al.* proposed a wearable system with stretchable electrodes for real-time vergence classification through optimized sensor placement and signal processing, enabling virtual reality (VR)–assisted treatment of convergence insufficiency (*16*). Nonetheless, susceptibility to experimental conditions rendering EOG outputs vulnerable to distortion remains a formidable challenge.

Advances in flexible electronics have enabled soft, miniaturized sensors for ocular monitoring, offering conformal and stable skin contact while minimizing discomfort during wear (*17*–*19*). Smart contact lenses exemplify this progress, offering continuous intraocular pressure tracking for glaucoma management (*20*, *21*), alongside emerging metabolic sensing applications (*22*–*24*). For eye tracking, scleral coil–based contact lenses are renowned for high angular resolution, yet their rigid coils and wired configuration necessitate ocular anesthesia, limiting clinical usability (*25*). Alternative strategies have introduced magnet-embedded contact lenses, whose position is detected by magnetoresistive sensors mounted on eyeglass frames (*26*), or battery-free radio frequency (rf)–tag lenses with frequency-swept readers to monitor signal variations (*27*). However, these approaches rely on the eyeglass frame as a fixed reference. Positional shifts between the frame and the head, as well as natural lens sliding during blinks, inevitably degrade accuracy. In contrast, flexible epidermal sensors overcome this limitation by maintaining a consistent coordinate system with head movements (*28*–*30*). It can also address limitations inherent in vision-based eye tracking systems (*31*–*33*). A flexible sensor array was designed for eye tracking, made of piezoelectric thin film attached to different parts of the temple (*34*, *35*). However, the small deflection detected in the temple area may be caused by other facial movements because the detection position is too far from the eyes. The large size and thickness lead to poor comfort. Moreover, the lack of a compact system-level design makes it difficult to apply in clinical diagnosis and establish a direct relationship with the disease.

Here, we propose a strategy for one-stop strabismus digital diagnosis using human-centric and artificial intelligence (AI)–integrated eye-wearable electronics, “Eyelectronics.” It uses deformation characteristics of the upper eyelid caused by eye movements to perform eye movement mapping. It is designed to be conformal to the eyelid and moves with the head, i.e., based on the Lagrangian coordinate system. A hierarchical-structured multidirectional strain-sensing (HMS) array is designed for in situ measurement of the eyelid strain changes. Featuring a disposable, ultralightweight, ultrathin, and breathable configuration, it promises comfort and mild-restricted measurement of the eyelid strain. The modeling-to-simulation-to-real (Model2Sim2Real) strategy of magnetic resonance imaging (MRI)–based eye modeling, finite element analysis (FEA), and digital image correlation (DIC) joint design establishes a prior correlation between eyelid deformation and eye movements, offering valuable insights for system design and effectively supporting diagnostic algorithm development. The physiology knowledge-driven algorithm is developed to achieve end-to-end strabismus diagnosis and address the poor generalizability caused by multiple diagnostic factors, with lightweight designs suitable for deployment on wearable edge devices. Clinical trials on patients with strabismus demonstrate that AI-integrated Eyelectronics provides a one-stop, wireless, and comfortable digital solution for simultaneous strabismus measurement and EOM function evaluation, thereby addressing the limitations of clinical diagnosis associated with multiple instruments and stepwise examinations of ocular alignment, EOM function, and deviation angle.

## RESULTS

### Strabismus measurement and EOM function evaluation via Eyelectronics

**Fig. 1.**
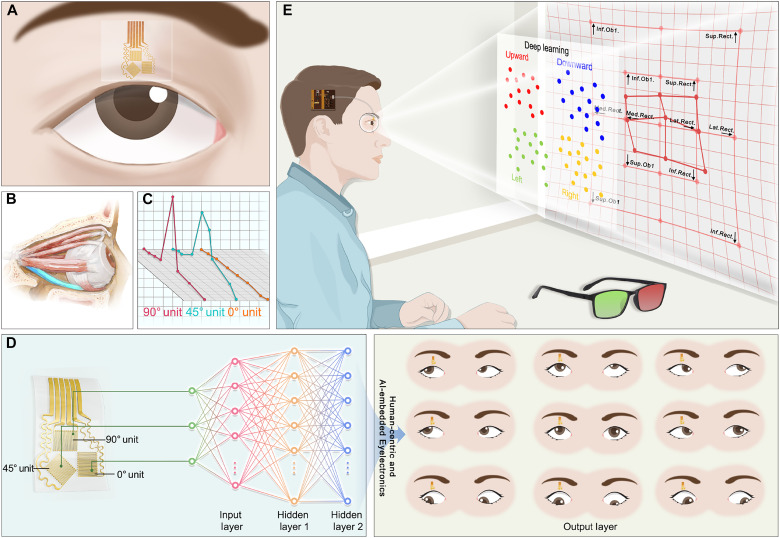
Schematic illustration of one-stop strabismus digital diagnosis via AI-integrated, skin-like, and wearable Eyelectronics. (**A**) The HMS array is conformally attached to the upper eyelid to measure eyelid strain caused by eye movements via morphological engineering. (**B**) Inferior rectus paralysis (marked in blue) causes the eye to deviate upward, resulting in eyelid deformation. (**C**) Typical signal example of measurement units arranged at angles of 0°/45°/90°. (**D**) One-stop strabismus digital diagnosis can be achieved through the end-to-end AI algorithm driven by physiology knowledge. (**E**) Eyelectronics features the eye-wearable HMS array, a head-mounted flexible circuit, and a Bluetooth-enabled device. Combining the Hess screen test, it can realize a wireless, comfortable, biocompatible, and one-stop digital solution for simultaneous strabismus measurement and EOM function evaluation. Illustrations in this figure were created using Photoshop.

As illustrated in Fig. 1, the skin-like and wearable Eyelectronics can serve for simultaneous strabismus measurement and EOM function evaluation by wireless and mild-restricted measurement of eyelid deformation caused by eye movements. In terms of devices, the HMS array achieves in situ measurement of eyelid strain by conformal attachment to the upper eyelid through ultrathin and breathable morphological design (Fig. 1A). As shown in Fig. 1B, abnormality of the EOM function causes the eye to be misaligned, causing the eyelids to become deformed. The three units (0°/45°/90°) are designed for multidirectional sensing, and their signals are shown in Fig. 1C. The current configuration is selected after considering both structural compactness and simple wiring. The relationship between eyelid deformation and eye movements may be nonlinear and highly abstract due to external factors like individual differences. Therefore, in terms of algorithms, an AI algorithm is developed to establish the corresponding relationship and achieve strabismus measurement while considering both accuracy and the memory requirements of wearable edge devices (Fig. 1D). The 0° and 90° units are mainly responsible for sensing eyelid deformation associated with horizontal (looking left and right; anatomically adduction toward the nose and abduction away from the nose) and vertical (looking upward and downward) eye movements, respectively. The 45° unit adds an additional channel to improve accuracy. Here, adduction and abduction refer to movements relative to the human eye, whereas in spatial terms, horizontal eye movements correspond to looking left and right.

Figure 1E illustrates the scenario of strabismus measurement and EOM function evaluation based on the AI-integrated Eyelectronics combined with the Hess screen test. The Hess screen test is used to determine the extent of muscle paralysis based on the size, shape, and position of the pattern consisting of the fixation points of the strabismus eye in all nine gaze directions. In terms of systems, Eyelectronics mainly contains a skin-like wearable HMS array, a head-mounted signal processing circuit, and a Bluetooth-enabled device. The compact design enables a wireless, comfortable, biocompatible, and one-stop digital solution for simultaneous strabismus measurement and EOM function evaluation. Detailed discussion can be found in the following sections.

### Design and fabrication of the Eyelectronics

**Fig. 2.**
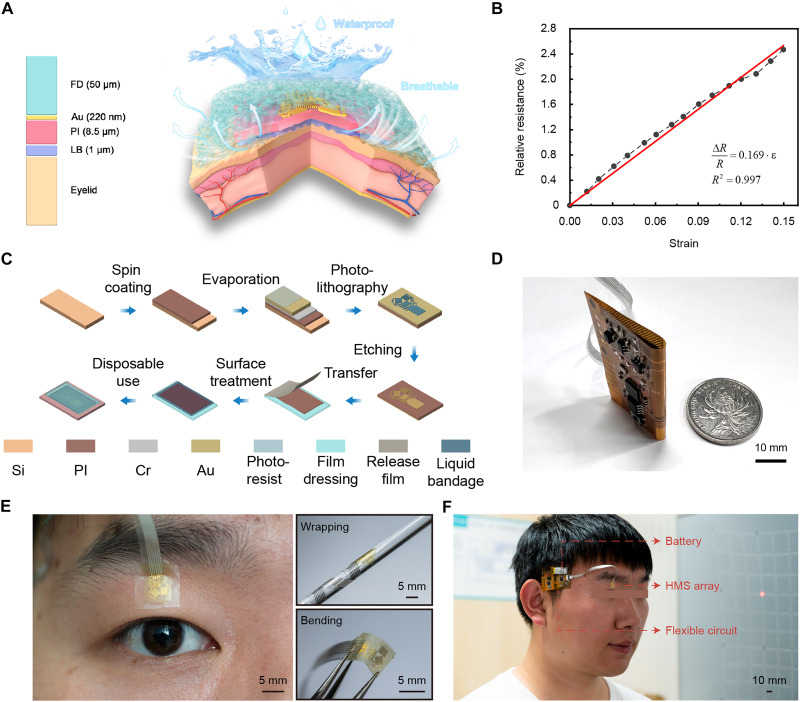
Design of the Eyelectronics. (**A**) Schematic illustration of the HMS array with a sectional view to show the structure, comprising a film dressing (FD), a strain-sensing part, and a layer of liquid bandage (LB) from top to bottom. The strain-sensing part consists of a metal sensing part (a Au layer) and a PI layer. The structure allows the array to be tightly attached to the eyelid as an epidermal tattoo-like patch without restricting eyelid deformation. It can be easily removed with the hybrid fixation of the dressing and liquid bandage. It also ensures the waterproofness and breathability of the array. (**B**) Linear relationship between the average value of the relative resistance change of the three units and the applied strain demonstrates that the sensitivity is 0.169 with the linear fit goodness of 0.997. (**C**) Fabrication flow chart of the HMS array enabling batch production and disposable medical consumables. (**D**) Flexible signal processing circuit folded to 180° is compared to one yuan coin, showing its compact size. Scale bar, 10 mm. (**E**) Ultrathin and flexible designs of the HMS array allow for conformal adhesion on eyelids. Optical image of the array (left), wrapped around a plastic tube with an outer diameter of 6 mm (top right), and in a bent state (bottom right). Scale bars, 5 mm. (**F**) Skin-like Eyelectronics mounted on a subject. Scale bar, 10 mm.

Eyelid skin, the thinnest skin in the human body, lacks subcutaneous fat and is prone to wrinkles, allergies, and edema. Therefore, flexible electronics on eyelids place higher requirements on flexibility, stretchability, breathability, and interface stability. Moreover, the core challenge of eyelid deformation measurement is that the mechanical properties of the device need to match it so as not to restrict its deformation. Figure 2A illustrates the HMS array containing a film dressing as the encapsulation layer, a strain-sensing part, and a layer of liquid bandage as the adhesion layer from top to bottom. The 50-μm-thick square transparent film dressing (Opsite, Smith & Nephew) provides full coverage and protection for the array like a piecrust. The strain-sensing part, like a filling, is fixed to the eyelid by it. The nanoporous dressing has waterproof, hypoallergenic encapsulation while permitting sweat vapor permeation without adverse effects on the skin, even with long-term use. Underneath the strain-sensing part, the liquid bandage (~1 μm in thickness) is sprayed between the part and the eyelid to achieve close contact with complex textures and wrinkles. This hybrid fixation balances adhesion strength with minimal motion restriction (low modulus) and allows painless removal. Furthermore, the lateral breathability of the bandage combined with the breathability of the dressing can ensure comfort during use. Photographs of eye movements while wearing the Eyelectronics are shown in fig. S1, demonstrating that the device barely restricts eyelid deformation caused by eye movements. The strain-sensing part consists of a metal sensing grid (Au layer, 220 nm in thickness, 6 mm by 6 mm in size) and a polyimide (PI) layer (8.5 μm in thickness) as a buffer layer. The PI layer can linearly transfer the eyelid strain to the strain-sensing part in a scaled-down manner based on the shear lag theory and protect the metal grid from damage (*29*). Calibration demonstrated a sensitivity of 0.169 with the linear fit goodness of 0.997, and the strain-sensing part could measure eyelid strain within 15% (Fig. 2B) and <0.59% sensitivity drift over cycles during the cycling test (fig. S2). The fabrication process of the HMS array is shown in Fig. 2C, which ensures ultrathin thickness, breathability, and no foreign body sensation. It can also be mass-produced and used as disposable medical consumables, overcoming the high-cost limitation of strabismus diagnosis.

Validation experiments were done to verify the rationality of the array response. When the eyes look upward or downward, the upper eyelid is compressed or stretched vertically. A similar thing happens when the eyes move horizontally. As a result, when a normal person makes eyes move upward or downward, only 45° and 90° units have signals, and the 0° unit barely has any signal (fig. S3). The situation is similar when eyes move horizontally. Figure S4 demonstrates the ability of the array to respond to eye movements of different amplitudes and frequencies. The amplitudes (~5° to 15°) and frequencies (~0.1 to 1 Hz) were selected to mimic the typical range of quasistatic or slow saccadic eye movements observed under normal physiological conditions. The specific range was chosen to ensure that the eyelid strain sensor experiences the deformation likely to occur during clinical scenarios.

The array resistance changes are measured by dividing the voltage in series with standard resistors (fig. S5). The analog-to-digital‌ converter converts the voltage into a digital signal, which is processed by the microcontroller unit and then transmitted to the Bluetooth device. The temperature compensation module reduces the temperature impact on the measurement. The flexible circuit can be powered by a battery, and the low-dropout regulator ensures the stability of the supply voltage. It can be bent 180° with a small curvature radius, showing the compact size (5 cm by 4 cm) compared to one yuan coin (Fig. 2D). The 2.48-g weight makes it completely stress-free to wear. Figure 2E shows that the array is fixed conformally to the eyelid via hybrid fixation. It can be wrapped around a tube with an outer diameter of 6 mm and bent 90° without being damaged. It interfaces with the circuit via an anisotropic conductive film (ACF), enabling modular replacement and disposable medical consumables. Figure 2F shows that the Eyelectronics is comfortably worn on the subject. Movie S1 records the process of eye tracking using the wearable system, and the 90° unit signal when looking upward. Movie S2 records the process of the array sensing strain and communicating via Bluetooth. Figure S6 shows the interface presentation of collecting resistance values of the HMS array with a smartphone.

### FEA of Eyelectronics monitoring with MRI 3D-reconstructed eye models

**Fig. 3.**
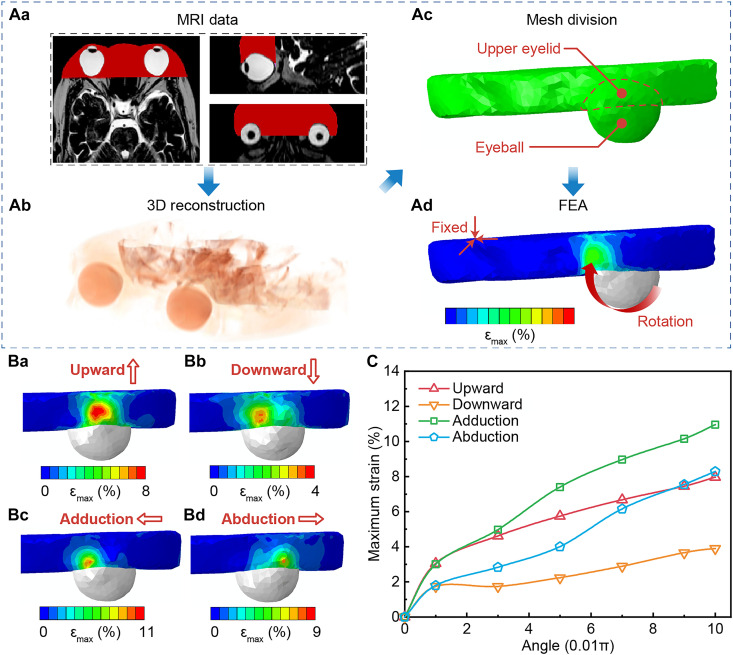
FEA of strain changes on the upper eyelid during eye movements. (**A**) FEA proposal. First, MRI data of a human head are obtained for FEA model reconstruction. Left, top right, and bottom right are the top view, left view, and front view of the eye region represented by the red region (**Aa**), respectively. Second, 3D reconstructions of the skull, eyeball, and upper eyelid are performed from the MRI data (**Ab**). Third, the region of interest was meshed, and the mesh density of the upper eyelid was refined appropriately (**Ac**). Last, eye movements cause strain changes on the eyelid (**Ad**). (**B**) Strain fields on the upper eyelid of four eyeball movements in directions of upward (**Ba**), downward (**Bb**), adduction (**Bc**), and abduction (**Bd**). (**C**) FEA results show that maximum values of eyelid strain during eye movements increase with the eye movement angle, verifying theoretically the correctness of the principle of eye tracking through measuring the eyelid strain.

Eye modeling and eyelid deformation simulation were performed to verify that eye tracking can be achieved through eyelid strain measurement (Fig. 3A). First, we performed three-dimensional (3D) modeling of the eyeball. To truly reflect the deformation of the eyelid during eye movement, we obtained a human head MRI image from the hospital for 3D modeling. Regions of the eyeball and the eyelid can be distinguished by the gray value differences on MRI images. Next, the 3D reconstruction model is imported into the FEA software to simulate eyelid deformation. For ease of analysis, the eyeball rotation center was set at the geometric center. The eyeball was controlled to rotate in four directions, with a maximum angle of 18°. This is because the eye movement range on the Hess screen test is generally 15°, and most naturally occurring human saccades have magnitudes of 15° or less (*36*).

The simulation results indicate that eye movement will cause eyelid deformation, so eye tracking can be achieved by Eyelectronics. The maximum strain ε_max_ on the upper eyelid surface is no more than 4% when looking downward (Fig. 3B). The maximum strains in the other three directions are not much different and do not exceed 11%. Hence, the strain change in the upper eyelid can be measured by the array. Furthermore, the location of the highest strain region on the eyelid changes with eye movements. Therefore, the array should be attached to the middle position in the horizontal direction, accounting for both adduction and abduction. Figure 3C shows that, as the eyeball rotation angle increases, the maximum strain of the eyelids also gradually increases. In other words, as the eyeball rotation angle increases, the array signal also increases. This theoretically verifies the principle of eye tracking through Eyelectronics and serves as a solid foundation for the physiology knowledge-driven AI algorithm.

### Verification of eyelid strain measurements of the Eyelectronics via the DIC method

**Fig. 4.**
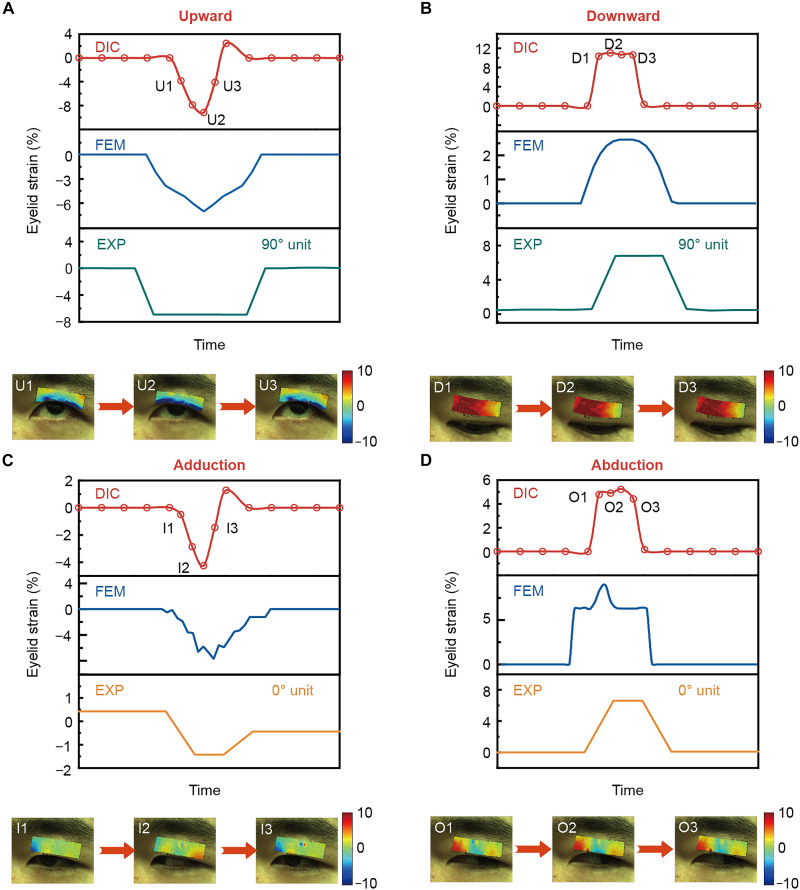
Eyelid strain measurement via DIC and comparison of the measured signal characterizations with those via the FEA and the HMS array. (**A** and **B**) The upper side shows the vertical strain changes of the eyelid obtained by DIC (red line), FEM (blue line), and the 90° unit of the HMS array (green line) as the eye looks upward and downward. The lower side shows images of the strain field changes measured by DIC. (**C** and **D**) The upper side shows the horizontal strain changes of the eyelid obtained by DIC (red line), FEM (blue line), and the 0° unit of the HMS array (orange line) as the eye undergoes adduction and abduction. The lower side shows images of the strain field changes measured by DIC. It can be seen that the waveform characteristics of the three signals are consistent, proving the feasibility of the Eyelectronics.

DIC serves as the benchmark for multidirectional eyelid strain measurement. The process is detailed in Materials and Methods. A speckle-patterned tattoo sticker replaced traditional methods to avoid adverse effects, whose preparation is detailed in Materials and Methods. Strain analysis revealed maximal deformation near the eyelid’s lower edge in the vertical direction (Fig. 4, A and B). Hence, the most suitable position of the array should be close to the lower edge. Considering the applicability to single or double eyelids and the extra space occupied by the dressing, the rectangular area is taken as the final attachment position, whose lower edge is ~3 mm from the eyelid’s lower edge (*37*). In addition, it should be attached to the eyelid middle in the horizontal direction according to the FEA.

The average strain at the attachment location is calculated and fitted to the curve. Figure 4 demonstrates vertical and horizontal eyelid strain patterns measured by DIC, FEA, and the array. Looking upward and then straight ahead first compresses the eyelid (negative 90° unit signal), followed by stretching (signal returning to baseline), while looking downward reverses this trend (Fig. 4, A and B). Horizontal adduction/abduction movements similarly induce compressive-tensile strains reflected by 0° unit signals (Fig. 4, C and D). All methods show consistent waveform shapes and directional polarity (positive/negative signals), validating the array’s ability to accurately measure multidirectional deformation. The Model2Sim2Real strategy of the FEA-DIC joint design provides a reference for sensor design, preparation, and adhesion positioning in practical settings. Signal polarity differences between 0° and 90° units enable eye movement classification and regression. As a result, Eyelectronics is capable of eye tracking by measuring the eyelid strain, thereby realizing the digital diagnosis of strabismus.

### Development of the AI algorithm for ocular motility examinations

**Fig. 5.**
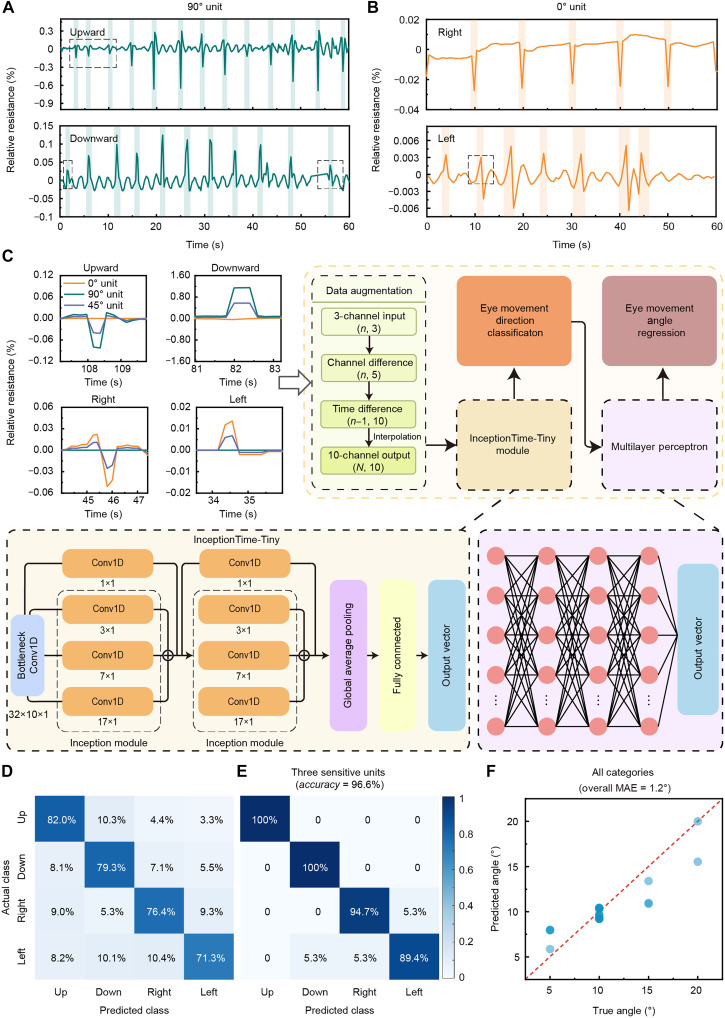
Accurate eye tracking via AI-integrated Eyelectronics. (**A**) Upward and downward vertical eye movements and the corresponding 90° unit signals. (**B**) Right and left horizontal eye movements and the corresponding 0° unit signals. The example signals marked by a black dashed box are difficult to accurately classify the direction of eye movements based on the measurement units in different directions and the polarity of the signal. (**C**) Flow of the InceptionTime-Tiny algorithm. Original signals of eye movements in four directions from 0°/45°/90° units are input into the physiology-driven algorithm after channel difference and time difference to realize eye movement direction classification, and then the angle regression is realized through an MLP network. (**D**) Accuracy of eye movement classification based on positive and negative signals from a single measurement unit. (**E**) Accuracy of eye movement classification based on the InceptionTime-Tiny algorithm. (**F**) Overall MAE of eye movement regression in the four directions is less than 1.2°.

Figure 5 (A and B) shows the results of continuous eye tracking in a simulated outpatient ocular motility examination. The 90° unit and 0° unit signals, corresponding to vertical and horizontal eye movements, respectively, were processed by the high-pass filter with a 0.5-Hz cutoff frequency for removing baseline wander. The 90° unit and the 0° unit have positive and negative signal differences in vertical and horizontal eye movements, respectively. However, some signals, such as the first few signals marked by a black dashed box in Fig. 5A, have small amplitudes and waveforms similar to those of noise, making it difficult to accurately classify eye movements. In addition, eyelid deformation may also be influenced by other various factors in actual use, such as eyeball shapes, attachment position errors, and environmental influences. Hence, eyelid strain features are difficult to extract through machine learning. The top-left part of Fig. 5C shows an example of signals from 0°/45°/90° units of the HMS array for eye movements in four directions. Based only on the polarity of the signal, eye movement classification has a relatively low accuracy of 77.4% (Fig. 5D). Therefore, a physiology-driven end-to-end AI algorithm is developed to realize accurate and efficient eye tracking.

Considering the distinctive features of the HMS array, designed specifically for mild-restricted, in situ multidirectional measurement of upper eyelid strain, we propose a specialized deep learning architecture tailored explicitly for efficient eye movement tracking. Leveraging the physiological signal properties uniquely captured by the array, we developed the lightweight InceptionTime-Tiny algorithm, an optimized variant inspired by the state-of-the-art InceptionTime architecture (*38*, *39*), seamlessly integrated into the Eyelectronics (Fig. 5C and note S1). It processes a 10-channel input obtained through channel difference and time difference from the initial three-unit signals. This differential approach effectively captures signal characteristics of different units and eyelid deformations of different time lengths, enhancing the algorithm’s discriminative capability.

The classification outputs are structured through four output channels within a compactly designed two-depth hierarchical module, each integrating 32 convolutional filters. Compressing 10-channel inputs into a 32D feature space helps reduce subsequent computational complexity. The selection of 32 dimensions is a collaborative consideration of model lightweighting and low-frequency eye movement signals (32 dimensions can already preserve enough principal components). Strategically designed kernel sizes of 3, 7, and 17 precisely target localized and global features related to eye movement onset, aligning closely with the biomechanical time-domain signals captured by the HMS array, corresponding to instantaneous eye movements, motion segments, and overall trends. Bottleneck layers ensure computational efficiency, with consistent padding maintaining dimensional stability throughout processing. Moreover, the more layers the Inception module has, the more abstract the extracted features become as eye movement classification can already be performed on the basis of the polarity of the signal. Therefore, two layers are selected. This architecture effectively captures intricate patterns within the data through its multiscale convolutional mechanism (*40*). After the classification task, we introduce a separate regression task to predict the eye movement angle. A simple multilayer perceptron (MLP) network is applied to each of the classified categories. These two tasks of classification and regression are trained and validated separately. This design, detailed in Materials and Methods, renders the algorithm relatively lightweight, making it theoretically ideal for deployment on wearable edge devices with limited memory capabilities.

The eye movement dataset was obtained by setting four directional test points on the screen at a fixed distance of 0.5 m and asking subjects to look at them. The distance between each adjacent test point represented 5°, and the maximum angle is 20° in one direction. For eye movement classification, 5000 pieces of data generated on the basis of 50% of the dataset were fed into the training model. The average classification accuracy was improved to 96.6% tested on the remaining 50% of the dataset, with 100% accuracy for vertical movements and 89% for looking left (Fig. 5E). Training metrics (loss, accuracy, and F1-score) are shown in fig. S7. Then, 80% of the dataset was fed into the MLP network, and the regression model was tested by the remaining 20% of the dataset. The overall mean absolute error (MAE) of eye movement regression in the four directions was finally less than 1.2° (Fig. 5F). The loss trends of regression in four directions are shown in fig. S8. These results demonstrate the algorithm clinical viability for precise strabismus measurement through robust classification-regression synergy, even with its lightweight design.

Furthermore, we have extended the dataset from the original four primary directions (up, down, right, and left) to include the remaining four oblique directions (up-left, up-right, down-left, and down-right), forming an eight-direction gaze dataset. In addition, blinking and facial expressions such as happiness, surprise, and anger were introduced into the dataset as the ninth category. The model was retrained accordingly, yielding a direction classification accuracy of 96.5% (fig. S9). The resulting misclassification rate of blinking and facial expressions is zero, indicating that the model does not classify expressions such as blinking or happiness as the direction of a particular eye movement, demonstrating strong robustness to facial motion confounders. We further analyzed the impact of small and large eye movements on the accuracy of direction classification. Four-direction classification shows that the model maintains a high accuracy of 94.4% (small saccades) and 92.3% (large saccades), confirming its ability to distinguish gaze directions for both subtle and large eye movements.

To further support this choice, we compared the InceptionTime-Tiny model with a classical KNN-DTW (*k*-nearest neighbors with dynamic time warping) method using the same eight-direction dataset. The KNN-DTW is a classical time-series classification approach that measures similarity between temporal sequences based on their optimal alignment in time. However, it lacks scalability and feature-learning capability compared with deep models. As shown in fig. S10, the KNN-DTW model achieved an overall accuracy of 78.4%, whereas our approach reached 96.5%, demonstrating its superior ability to generalize across gaze directions while remaining computationally lightweight and suitable for edge deployment.

### Clinical trials of strabismus evaluation on patients

**Fig. 6.**
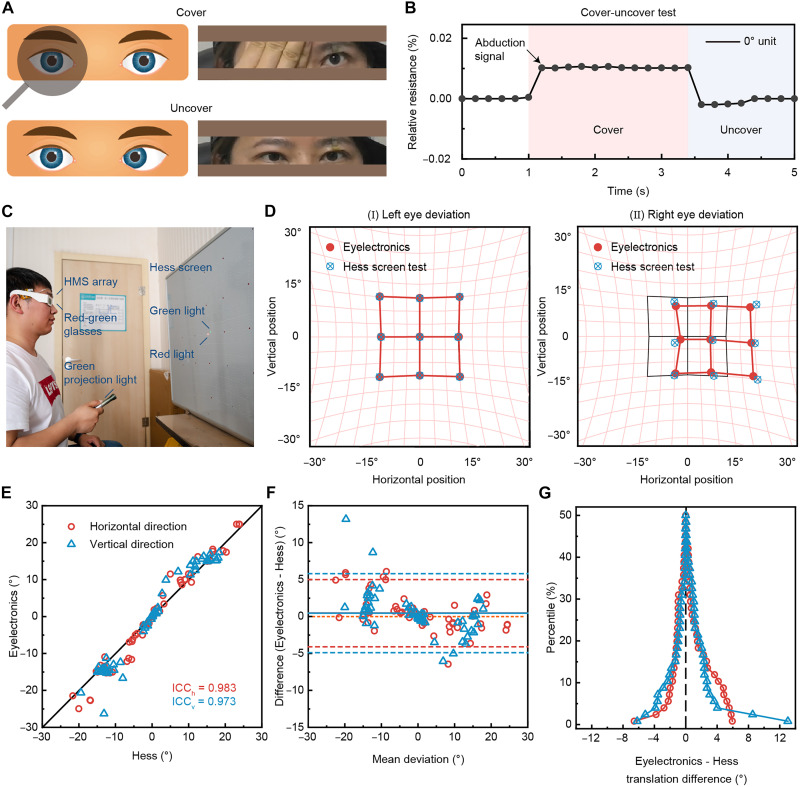
One-stop digital diagnosis of strabismus with the AI-integrated, skin-like, and wearable Eyelectronics. (**A**) Schematic diagram of the cover-uncover test and photograph of the eye movement in a patient with left eye esotropia. (**B**) Results of the cover-uncover test with the Eyelectronics. (**C**) Photograph of the Hess screen test using the Eyelectronics. (**D**) Comparisons of the standard Hess screen test results and the test results using the Eyelectronics from the left eye (normal eye) and the right eye (strabismic eye). (**E**) Correlation of ocular deviations of all nine cardinal positions between the Hess screen test and the test with the Eyelectronics. The ICCs for all nine horizontal and vertical deviations are 0.983 and 0.973, respectively. (**F**) Mean difference plot used to evaluate the agreement between two tests. Horizontal (0.47°, red solid line) and vertical (0.46°, blue solid line) mean differences are close to zero. The 95% LoAs are wider for vertical deviations (blue dashed line) compared with horizontal deviations (red dashed line). (**G**) Folded empirical cumulative distribution plots for illustration of the ranked differences between the two tests. The medians (peaks) of both horizontal (red) and vertical (blue) differences are centered over zero, indicating no systematic bias between the two tests. The narrower vertical peak indicates that the horizontal agreement is slightly closer than the vertical agreement. Illustrations in (A) were created using Photoshop.

The applicability verification of the cover-uncover test using the Eyelectronics was carried out. For patients with strabismus, cover testing is the clinical standard for the objective method for determining the presence, type, and magnitude of strabismus. The cover-uncover test is generally performed first, which is useful to identify a tropia and differentiate it from a phoria. The test is usually done by using a translucent occluder to cover one eye (Fig. 6A). The process and diagnostic criteria of the cover-uncover test are detailed in Materials and Methods. A patient with left eye esotropia underwent a cover-uncover test using the Eyelectronics. The left eye turned to the temporal side when the right eye was covered. After the right eye was uncovered, the left eye turned to the nasal side again. As shown in Fig. 6B, the abduction signal could be detected when the right eye was covered. When the abduction signal disappeared, it meant that the left eye deviated again after the right eye was uncovered. It can be inferred that the subject may have esotropia in the left eye, and the right eye is the fixating eye.

Combined with the Hess screen test, the Eyelectronics can realize one-stop strabismus measurement and EOM function evaluation. For incomitant strabismus, the ocular deviation varies with gaze direction. The Hess screen test is required to assess eye movements and detect muscle imbalances (Fig. 6C). The basic process of the Hess screen test is detailed in Materials and Methods. Movie S3 records the whole process of the Hess screen test. The test using the Eyelectronics does not require the patient’s participation in position marking and avoids the resulting diagnostic errors. Given the individual variations between patients with strabismus and healthy participants, the doctor should perform an initial calibration before conducting the test using the Eyelectronics. This entails acquiring five time-series signal data points per cardinal position of gaze. Subsequently, the InceptionTime-Tiny algorithm is fine-tuned through few-shot learning to ultimately generate the test results.

The human study comprised a prospectively enrolled cohort of seven participants: six clinically confirmed patients with strabismus and one healthy control. Patient demographics and strabismus types are summarized in table S1. The cohort covers a broad range of ages (18 to 52 years), both sexes, and different eyelid phenotypes (single and double eyelids), encompassing the most common strabismus patterns. The results of the standard Hess screen test and the test with the wearable system for the healthy participant are shown in fig. S11. It can be seen that the two tests have good consistency. Figure 6D shows the results for a strabismus subject with medial rectus paralysis in the right eye. The test was conducted six times, and the average was taken as the final result. First, analyzing the shape, the test results of both eyes are asymmetrical, so the patient has paralytic strabismus. Second, analyzing the size, the area containing fixation points of the right eye is smaller, so the right eye is paralyzed. Third, analyzing the position, the fixation point of the right eye is deviated to the right compared to the nine cardinal positions of gaze, which indicates that the right eye is exotropic. Last, this patient can be diagnosed with medial rectus paralysis of the right eye. Medial rectus paralysis in the right eye specifically involves the inability of the medial rectus muscle to contract effectively, resulting in the affected eye deviating in abduction. Therefore, digital diagnosis of strabismus can be achieved by analyzing the symmetry, size, and position of the Hess screen results through threshold condition judgment and image visualization. The Hess screen results of the affected eye in other patients, together with their corresponding tests obtained using the wearable system, are presented in fig. S12. Figure S13 shows the typical results of the Hess screen test for different EOM paralyses, taking the right eye as an example.

To further assess the consistency between the wearable system and the conventional Hess screen test across all patients, intraclass correlation coefficient (ICC) analysis was performed. The ICC for all nine horizontal deviations is 0.983, 95% confidence interval (CI) (0.972, 0.990); the ICC for the vertical deviations is 0.973, 95% CI (0.956, 0.984) (Fig. 6E); and the ICC for the total deviation is 0.978, 95% CI (0.969, 0.985). Substantial agreement can be found for all nine cardinal positions between the test with the Eyelectronics and the Hess screen test. Furthermore, the difference between the two tests varied with the size of ocular deviation is evaluated using Bland-Altman plots (Fig. 6F). The mean differences are close to zero for both directions, as well as the 95% limits of agreement (LoAs) are similar for horizontal [mean 0.47° LoA (−4.1°, 5.0°)] and vertical [mean 0.46° LoA (−4.9°, 5.8°)] directions. Hence, there is no significant proportional error between the two tests. As a complementary representation, folded empirical distribution plots for both the horizontal and vertical ranked differences are constructed (Fig. 6G). The peaks representing the median are close to zero in both horizontal and vertical distributions, indicating that the test with the wearable system is unbiased compared with the Hess screen test. The horizontal distribution is slightly narrower than the vertical one, indicating that the two tests are in closer agreement for horizontal deviations.

The few-shot adaptation was quantified using data from a randomly selected patient with six Hess screen recordings. The model was fine-tuned with two to five labeled samples and validated on the remaining recordings (fig. S14). Maximum MAEs decreased from 3.8° to 2.0° as training samples increased, with horizontal and vertical biases converging toward clinical Hess screen values (fig. S15). Each gaze point required ~5-s fixation, and a full few-shot calibration for a new subject could be completed within ~5 min.

Stratified analyses based on Bland-Altman plots for each direction were conducted to evaluate the potential effects of age (<35 versus ≥35 years), sex, and eyelid phenotype on measurement performance, reporting the bias and LoA with 95% confidence bands (fig. S16 and table S2). The bias values in both directions were close to zero, indicating minimal systematic error. The 95% LoAs were slightly wider intervals observed for older participants and single eyelid participants. Sex-based differences could be negligible. It indicated that measurement performance was consistent across age, sex, and eyelid phenotype subgroups, demonstrating the robustness and generalizability of the proposed system. Furthermore, the CIs for both MAE and directional accuracy at each gaze position were analyzed. Across all participants, the mean MAE values ranged from 1.0° to 4.3° depending on gaze position, with the central fixation showing the smallest error (1.0° ± 0.4°) and larger deviations observed at the up-left positions (table S3). Directional accuracy analysis showed that both horizontal and vertical biases were generally centered near zero (typically within ±1° to 2°), with no systematic over- or underestimation trends. Subgroup analysis further demonstrated consistent performance across age, sex, and eyelid phenotype (tables S4 to S6). As a result, it can be concluded that Eyelectronics can realize digital diagnosis of strabismus based on the Hess screen test, avoiding errors caused by the doctor’s subjective judgment and patient’s participation.

## DISCUSSION

In conclusion, a solution for one-stop strabismus digital diagnosis based on the skin-like and wearable Eyelectronics is proposed to address the core issues of multiple instruments, stepwise examinations, low diagnostic objectivity, poor pediatric compliance, and high cost in clinical strabismus diagnosis by synergizing advancements in AI and flexible electronics. It enables simultaneous strabismus measurement and EOM function evaluation while ensuring minimal physical and cooperation burden. The HMS array is designed to be conformally attached to the eyelid with only mild restriction by morphological engineering, achieving in situ multidirectional measurement of upper eyelid strain changes. The FEA of eye movement based on MRI data establishes a prior correlation between eyelid deformation and eye movements. In addition, using the DIC method enables the quantification of eyelid strain fields induced by eye movements as a reference, offering valuable insights for sensor design and effectively supports diagnostic algorithm development. The physiology knowledge-driven InceptionTime-Tiny algorithm is optimized for lightweight design based on eye movement signal characteristics, making it theoretically ideal for deployment on wearable devices. The AI-integrated Eyelectronics achieves robust eye tracking with a four-direction classification accuracy of 96.6% and an angle accuracy of 1.2°. The algorithm shows potential for future interpretability as distinct signal patterns from multiple sensing units consistently align with horizontal and vertical eye movements, supported by DIC, finite element method (FEM), and experimental analyses, thereby enhancing its clinical reliability and transparency. Clinical validation via cover-uncover and Hess screen tests confirmed diagnostic agreement. Enabled by the integration of AI and human-centric flexible electronics, the Eyelectronics overcomes the limitations of conventional diagnosis that rely on multiple instruments and stepwise examinations. It lays the foundation for transforming strabismus diagnosis and treatment from experience-based to data-driven paradigms, providing critical quantitative insights for surgical planning and personalized intervention.

Furthermore, synergizing with biomechanical models of EOMs, the system holds promise in advancing precision medicine approaches for surgical interventions targeting paralytic strabismus. However, the rapid verification of wearable devices through clinical trials to achieve medical-grade monitoring capabilities still faces the difficulties of insufficient data and insufficient sample size. The integration of MRI modeling, FEA, and flexible electronics into a Model2Sim2Real framework offers a comprehensive digital pathway to accelerate device iteration. It can be generated through physical-electrical modeling of wearable medical devices and simulation of the real medical process based on batch simulation and AI algorithms. Collectively, these advancements underscore the versatility and utility of the wearable system, paving the way for its integration into various clinical and technological settings.

## MATERIALS AND METHODS

### Fabrication of the strain-sensing array

Mature photolithography technology helps the array to be rapidly mass-produced with high manufacturing precision and strong batch stability. The fabrication process started with the preparation of PI with a thickness of 8.5 μm by spin coating on a silicon wafer at a speed of 3000 rpm. Second, Cr and Au were sequentially deposited on PI with thicknesses of 15 and 220 nm, respectively. Third, the patterned metal structure on the PI substrate could be obtained by photolithography and etching of the Cr-Au layer using the designed array mask. After a transfer printing step, the patterned sensing layer was encapsulated with a 50-μm-thick transparent film dressing, and the HMS array was obtained. The array was connected to the signal processing circuit with an ACF. Last, the overall thickness of the array was about 60 μm. When in use, the release film was tore off from the array and the liquid bandage was sprayed. Last, the array is attached to the eyelid, and the liquid bandage is allowed to solidify.

### Characterization of the strain-sensing array

The sensor calibration steps are as follows. First, the HMS array was attached to a polydimethylsiloxane (PDMS) stretch piece through the liquid bandage. Then, the stretch piece was gradually tensile loaded to a maximum strain of 15% by a tensile testing machine (fig. S2A). Five replicate experiments were performed. The linear relationship between applied strain and the relative resistance change (Fig. 2B) demonstrated that the sensitivity of the flexible strain sensor was 0.169 with a measurement range of up to 15%. Furthermore, the cycling test was performed three times by the tensile testing machine. The sensitivities of the array were 0.169, 0.169, 0.169, 0.169, 0.168, and 0.168, respectively (fig. S2B).

### 3D reconstruction model

The steps for 3D reconstruction based on human head MRI images are as follows. First, the MRI images from Beijing Tongren Hospital were imported into Mimics (version 19.0, Materialize, Leuven, Belgium), and the contrast and brightness were adjusted to enhance the visibility of the skull, eyeball, and upper eyelid. Next, representative slices that cover the entire region of interest were selected. Masks for the different structures were created using automatic thresholding techniques. These masks were manually refined with editing tools to ensure accuracy. Then, the 3D model was generated using the calculate 3D function, which was optimized by applying smoothing and simplification tools to refine the surfaces and structures. Last, the final model was exported in the STL file format for the mechanical simulation.

### Mechanical simulation

The FEA commercial software ABAQUS (Analysis User’s Manual 2019) was used to simulate the strain of the upper eyelid. Considering that Young’s modulus of the skull was several orders of magnitude larger than that of the upper eyelid and eyeball, the upper surface of the skull part was set as a fixed support to avoid the phenomenon of stress concentration in the connected part between the eyelid and skull. Because the eyelids exert little restraint on the eyeball, the eyeball deformation caused by the eyelids can usually be ignored. Hence, the eyeball was regarded as a rigid body. The model was a pure rotation of the eyeball in the eye socket. The eyeball surface was set as the master surface of the contact, whereas the eyelid surface was set as the slave surface. Because of the lubrication of the tear layer, the friction between the eyeball and the eyelid can be negligible. Hence, the tangential behavior was set to no friction, and the normal behavior was set to hard contact. For analysis of the eyelid deformation, the mesh density of the upper eyelid was refined appropriately. The eyelid was modeled by hexahedron elements (C3D4H), which were suitable for large deformation analysis of eyelids and helped improve stability and computational efficiency. The skull and the eyeball were modeled by the discrete rigid part. An elastic material model was used to model the eyelid with a Young’s modulus of 0.04 MPa and a Poisson ratio of 0.3.

### Speckle tattoo patch preparation

Black circular spots of a certain density and a certain diameter were randomly distributed in an area the size of the upper eyelid through MATLAB as a speckle pattern. Multiple sets of generated speckle pattern images were recorded and saved. The image was reversed and printed on the glossy side of the tattoo paper. The protective layer of the adhesive film was peeled off and carefully placed over the printed side of the tattoo paper. Before applying the tattoo sticker, the eyelid was gently cleaned with warm water to remove oil. The tattoo was placed face down on the skin, and the backing paper was dampened with a wet sponge and pressed gently for about 30 s. The backing paper was gently lifted from the tattoo to ensure that it adheres fully to the skin (fig. S17). Last, the strain field of the upper eyelid can be measured on the basis of the DIC method. After the measurement, a mild, eye-safe makeup remover on a cotton swab was used to dissolve the tattoo.

### Procedures for the strain field on the upper eyelid

The obtained speckle images of the deformed upper eyelid captured by the charge-coupled device (CCD) camera (SP-5000C-USB, JAI) were processed using the DIC technique by Ncorr, an open-source software tool in the MATLAB platform (The MathWorks Inc., USA). Specifically, the procedures to perform the 2D-DIC measurement were as follows. First, the original image before eye movements were captured with the CCD camera with a 50-mm focal length lens, which was used as the reference image for DIC calculation. Next, a series of eyelid speckle images was continuously captured while the eyes were looking upward, downward, left, and right. Then, the strain field could be calculated by the local least-squares fitting method based on the results of the displacement field.

### Training methodology for the AI algorithm for eye movement classification and regression

In our study, we developed the InceptionTime-Tiny algorithm based on the Tsai platform, which was designed to analyze complex time-series data effectively. The training was executed under Python 3.9 and PyTorch 2.1 with the Adam optimizer. We set an adaptive learning rate starting at 0.001 and adjusted dynamically over 20 epochs to ensure effective learning without overfitting. The training process incorporates the rectified linear unit (ReLU) activation function and a dropout mechanism to enhance model generalization and prevent overfitting. This approach ensured that the algorithm was not only accurate but also robust against data that had not previously been encountered.

For the classification task, the algorithm was trained to classify the eye movement direction, with evaluation metrics such as accuracy, precision, and recall. Following the classification, a separate training phase was conducted for the regression task, where a simple MLP network was trained for predicting the eye movement angle. During training, the regression model was fitted only to the samples corresponding to the predicted categories. Each task was evaluated independently, ensuring that both the classification and regression performances were optimized. This algorithm supports input and output of any number of channels, so it can switch between predicting four directions (up, down, right, and left) or eight directions classification (the same four plus up-left, up-right, down-left, and down-right). In the regression task, the angular regression of a single direction can be converted into the prediction of spatial coordinate points by establishing a loss function of spatial distance.

The InceptionTime-Tiny algorithm comprises 65,156 parameters, structured to balance complexity with computational efficiency. Its initial bottleneck layer contains 32 filters of size 10 by 1, and subsequent layers use kernels sized 17, 7, and 3 to process data at varying scales. This design renders the algorithm relatively lightweight, making it theoretically ideal for deployment on devices with limited memory capabilities, such as wearable technology. The total size is around 254 kB, assuming each parameter is stored as a 32-bit float, which aligns well with the memory constraints of modern wearable devices. The regression model consists of a simple MLP network with four layers, where the output is a continuous value representing the eye movement angle. This MLP model contains 44,033 parameters with a total size of ~172 kB, further demonstrating the model’s compactness and efficiency, contributing to the overall performance.

### Basic process of the Hess screen test

The Hess screen is composed of horizontal and vertical lines, and the width between any two adjacent lines is equivalent to 5° when the patient is half a meter away. During the Hess screen test, the subject wears red and green lenses for the watching eye and the subject eye, respectively. The subject’s eyes are facing the red light in the center, and the green projection light is held to track the red light controlled by the examiner on the screen so that the two lights overlap. At this time, the patient’s eyes are injected with red and green visual targets in the fovea of the macula. The separation distance between these two visual targets is equal to the subject’s objective strabismus angle. Next, the point light sources are lit up on the nine cardinal positions of gaze in sequence and the above inspection is repeated. Then, the fixating eye is changed, and the strabismus angle is checked when the other eye is fixating. Last, the examination results are analyzed to determine the strabismus type, strabismus angle, and paralyzed muscles. As a comparison, the gaze position of the eyes can be directly recorded with the Eyelectronics without the green projection light.

### In vivo experiment with human subjects

The eye-tracking study in the cover-uncover test and the Hess screen test involved eight volunteers, and the study was conducted by following the approved Institutional Review Board protocol (no. TREC2025-KY002) at the Beijing Tongren Hospital. Strabismus subject 1: gender F, diagnosed with esotropia in the left eye. Strabismus subject 2: gender M, diagnosed with paralysis strabismus with medial rectus paralysis of the right eye. Strabismus subject 3: gender F, diagnosed with hypertropic esotropia in the left eye. Strabismus subject 4: gender M, diagnosed with hypotropic exotropia in the left eye. Strabismus subject 5: gender M, diagnosed with esotropia in the left eye. Strabismus subject 6: gender M, diagnosed with esotropia in the right eye. Strabismus subject 7: gender F, diagnosed with hypertropic esotropia in the right eye. Healthy participant 1: gender M, no relevant health conditions. Before the in vivo study, all subjects agreed to the study procedures and provided signed consent forms.

## References

[1] M. Dysli, F. C. Fierz, D. Rappoport, T. S. Meier, K. Landau, C. J. Bockisch, K. P. Weber, Divergence bias in Hess compared to Harms screen strabismus testing. Strabismus 29, 1–9 (2021). 33591220 10.1080/09273972.2020.1871382

[2] T. Wygnanski-Jaffe, B. J. Kushner, A. Moshkovitz, M. Belkin, O. Yehezkel, CureSight Pivotal Trial Group, An eye-tracking-based dichoptic home treatment for amblyopia: A multicenter randomized clinical trial. Ophthalmology 130, 274–285 (2023). 36306974 10.1016/j.ophtha.2022.10.020

[3] P. H. Yeh, C. H. Liu, M. H. Sun, S. C. Chi, Y. S. Hwang, To measure the amount of ocular deviation in strabismus patients with an eye-tracking virtual reality headset. BMC Ophthalmol. 21, 246 (2021). 34088299 10.1186/s12886-021-02016-z PMC8178882

[4] N. M. Bakker, B. A. Lenseigne, S. Schutte, E. B. Geukers, P. P. Jonker, F. C. van der Helm, H. J. Simonsz, Accurate gaze direction measurements with free head movement for strabismus angle estimation. IEEE Trans. Biomed. Eng. 60, 3028–3035 (2013). 23399951 10.1109/TBME.2013.2246161

[5] R. L. Thorisdottir, J. Sundgren, R. Sheikh, J. Blohme, B. Hammar, S. Kjellstrom, M. Malmsjo, Comparison of a new digital KM screen test with conventional Hess and Lees screen tests in the mapping of ocular deviations. J. AAPOS 22, 277–280.e6 (2018). 29852255 10.1016/j.jaapos.2018.02.007

[6] O. Yehezkel, M. Belkin, T. Wygnanski-Jaffe, Automated diagnosis and measurement of strabismus in children. Am. J. Ophthalmol. 213, 226–234 (2020). 31887281 10.1016/j.ajo.2019.12.018

[7] N. Nixon, P. B. M. Thomas, P. R. Jones, Feasibility study of an automated Strabismus screening Test using Augmented Reality and Eye-tracking (STARE). Eye (Lond.) 37, 3609–3614 (2023). 37142780 10.1038/s41433-023-02566-0 PMC10686399

[8] Y. Iwata, T. Handa, H. Ishikawa, Objective measurement of nine gaze-directions using an eye-tracking device. J. Eye Mov. Res. 13, 1–8 (2020). 10.16910/jemr.13.6.4 PMC8015013 33828814

[9] O. Zrinscak, I. Grubisic, K. Skala, J. Skunca Herman, T. Kriz, R. Ivekovic, Computer based eye tracker for detection of manifest strabismus. Acta Clin. Croat. 60, 683–694 (2021). 35734485 10.20471/acc.2021.60.04.16 PMC9196215

[10] K. P. Weber, D. Rappoport, M. Dysli, T. Schmuckle Meier, G. B. Marks, C. J. Bockisch, K. Landau, H. G. MacDougall, Strabismus measurements with novel video goggles. Ophthalmology 124, 1849–1856 (2017). 28728924 10.1016/j.ophtha.2017.06.020

[11] E. Orduna-Hospital, L. Maurain-Orera, C. Lopez-de-la-Fuente, A. Sanchez-Cano, Hess lancaster screen test with eye tracker: An objective method for the measurement of binocular gaze direction. Life (Basel) 13, 668 (2023). 36983824 10.3390/life13030668 PMC10054291

[12] A. Al-Rahayfeh, M. Faezipour, Eye tracking and head movement detection: A state-of-art survey. IEEE J. Transl. Eng. Health Med. 1, 2100212 (2013). 27170851 10.1109/JTEHM.2013.2289879 PMC4839304

[13] C. Belkhiria, A. Boudir, C. Hurter, V. Peysakhovich, EOG-based human-computer interface: 2000-2020 review. Sensors (Basel) 22, 4914 (2022). 35808414 10.3390/s22134914 PMC9269776

[14] C. T. Lin, W. L. Jiang, S. F. Chen, K. C. Huang, L. D. Liao, Design of a wearable eye-movement detection system based on electrooculography signals and its experimental validation. Biosensors (Basel) 11, 343 (2021). 34562933 10.3390/bios11090343 PMC8471050

[15] S. Ban, Y. J. Lee, S. Kwon, Y. S. Kim, J. W. Chang, J. H. Kim, W. H. Yeo, Soft wireless headband bioelectronics and electrooculography for persistent human-machine interfaces. ACS Appl. Electron. Mater. 5, 877–886 (2023). 36873262 10.1021/acsaelm.2c01436 PMC9979786

[16] S. Mishra, Y. S. Kim, J. Intarasirisawat, Y. T. Kwon, Y. Lee, M. Mahmood, H. R. Lim, R. Herbert, K. J. Yu, C. S. Ang, W. H. Yeo, Soft, wireless periocular wearable electronics for real-time detection of eye vergence in a virtual reality toward mobile eye therapies. Sci. Adv. 6, eaay1729 (2020). 32201718 10.1126/sciadv.aay1729 PMC7069716

[17] Z. Wang, N. Shi, Y. Zhang, N. Zheng, H. Li, Y. Jiao, J. Cheng, Y. Wang, X. Zhang, Y. Chen, Y. Chen, H. Wang, T. Xie, Y. Wang, Y. Ma, X. Gao, X. Feng, Conformal in-ear bioelectronics for visual and auditory brain-computer interfaces. Nat. Commun. 14, 4213 (2023). 37452047 10.1038/s41467-023-39814-6 PMC10349124

[18] X. Feng, Y. Huang, H. Jiang, D. Ngo, A. J. Rosakis, The effect of thin film/substrate radii on the Stoney formula for thin film/substrate subjected to nonuniform axisymmetric misfit strain and temperature. J. Mech. Mater. Struct. 1, 1041–1053 (2006).

[19] M. A. Brown, A. J. Rosakis, X. Feng, Y. Huang, E. Üstü\ndag, Thin film/substrate systems featuring arbitrary film thickness and misfit strain distributions. Part II: Experimental validation of the non-local stress/curvature relations. Int. J. Solid. Struct. 44, 1755–1767 (2007).

[20] W. Liu, Z. Du, Z. Duan, L. Li, G. Shen, Neuroprosthetic contact lens enabled sensorimotor system for point-of-care monitoring and feedback of intraocular pressure. Nat. Commun. 15, 5635 (2024). 38965218 10.1038/s41467-024-49907-5 PMC11224243

[21] H. An, X. Wang, Z. Liao, L. Zhang, H. Zhao, Y. Yang, J. Song, Y. Ma, LC contact lens sensor for ultrasensitive intraocular pressure monitoring. NPJ Flex. Electron. 8, 53 (2024).

[22] J. Zhu, L. Yang, Q. Yang, Y. Huang, Y. Li, Y. He, X. Yang, Z. Yang, Y. Jiao, W. Wei, Y. Chen, X. Feng, Kirigami-inspired breathable smart contact lens for wireless monitoring of corneal hypoxia and microenvironment. Adv. Healthc. Mater. 14, e2402148 (2025). 40417862 10.1002/adhm.202402148 PMC12264840

[23] W. Park, H. Seo, J. Kim, Y. M. Hong, H. Song, B. J. Joo, S. Kim, E. Kim, C. G. Yae, J. Kim, J. Jin, J. Kim, Y. H. Lee, J. Kim, H. K. Kim, J. U. Park, In-depth correlation analysis between tear glucose and blood glucose using a wireless smart contact lens. Nat. Commun. 15, 2828 (2024). 38565532 10.1038/s41467-024-47123-9 PMC10987615

[24] S. K. Kim, G. H. Lee, C. Jeon, H. H. Han, S. J. Kim, J. W. Mok, C. K. Joo, S. Shin, J. Y. Sim, D. Myung, Z. Bao, S. K. Hahn, Bimetallic nanocatalysts immobilized in nanoporous hydrogels for long-term robust continuous glucose monitoring of smart contact lens. Adv. Mater. 34, e2110536 (2022). 35194844 10.1002/adma.202110536 PMC10782562

[25] C. Ghosh, A. Mastrangelo, M. Karkhanis, A. Deshpande, A. Banerjee, H. Kim, C. H. Mastrangelo, Low-profile induced-voltage distance ranger for smart contact lenses. IEEE Trans. Biomed. Eng. 68, 2203–2210 (2021). 33232221 10.1109/TBME.2020.3040161

[26] A. Tanwear, X. Liang, Y. Liu, A. Vuckovic, R. Ghannam, T. Bohnert, E. Paz, P. P. Freitas, R. Ferreira, H. Heidari, Spintronic sensors based on magnetic tunnel junctions for wireless eye movement gesture control. IEEE Trans. Biomed. Circuits Syst. 14, 1299–1310 (2020). 32991289 10.1109/TBCAS.2020.3027242

[27] H. Zhu, H. Yang, S. Xu, Y. Ma, S. Zhu, Z. Mao, W. Chen, Z. Hu, R. Pan, Y. Xu, Y. Xiong, Y. Chen, Y. Lu, X. Ning, D. Jiang, S. Yuan, F. Xu, Frequency-encoded eye tracking smart contact lens for human-machine interaction. Nat. Commun. 15, 3588 (2024). 38678013 10.1038/s41467-024-47851-y PMC11055864

[28] X. Feng, Y. Huang, A. J. Rosakis, Stresses in a multilayer thin film/substrate system subjected to nonuniform temperature. J. Appl. Mech. 75, 021022 (2008).

[29] Y. Chen, B. Lu, Y. Chen, X. Feng, Biocompatible and ultra-flexible inorganic strain sensors attached to skin for long-term vital signs monitoring. IEEE Electron Device Lett. 37, 496–499 (2016).

[30] Y. Chen, S. Lu, S. Zhang, Y. Li, Z. Qu, Y. Chen, B. Lu, X. Wang, X. Feng, Skin-like biosensor system via electrochemical channels for noninvasive blood glucose monitoring. Sci. Adv. 3, e1701629 (2017). 29279864 10.1126/sciadv.1701629 PMC5738229

[31] D. Vera Anaya, T. He, C. Lee, M. R. Yuce, Self-powered eye motion sensor based on triboelectric interaction and near-field electrostatic induction for wearable assistive technologies. Nano Energy 72, 104675 (2020).

[32] L. Massin, F. Seguin, V. Nourrit, E. Daniel, J.-L. de Bougrenet de la Tocnayet, C. Lahuec, Smart contact lens applied to gaze tracking. IEEE Sens. J. 21, 455–463 (2021).

[33] B. Gao, Z. He, B. He, Z. Gu, Wearable eye health monitoring sensors based on peacock tail-inspired inverse opal carbon. Sens. Actuators B Chem. 288, 734–741 (2019).

[34] N. I. Kim, J. Chen, W. Wang, M. Moradnia, S. Pouladi, M. K. Kwon, J. Y. Kim, X. Li, J. H. Ryou, Highly-sensitive skin-attachable eye-movement sensor using flexible nonhazardous piezoelectric thin film. Adv. Funct. Mater. 31, 2008242 (2021).

[35] N. I. Kim, J. Chen, W. Wang, J. Y. Kim, M. K. Kwon, M. Moradnia, S. Pouladi, J. H. Ryou, Skin-attached arrayed piezoelectric sensors for continuous and safe monitoring of oculomotor movements. Adv. Healthc. Mater. 13, e230581 (2024). 10.1002/adhm.202303581 38386698

[36] K. Pettersson, J. Tervonen, J. Heininen, J. Mäntyjärvi, Head-area sensing in virtual reality: Future visions for visual perception and cognitive state estimation. Front. Virtual Real. 5, 1423756 (2024).

[37] T. Y. Lu, K. Kadir, W. C. Ngeow, S. A. Othman, The prevalence of double eyelid and the 3D measurement of orbital soft tissue in malays and chinese. Sci. Rep. 7, 14819 (2017). 29093554 10.1038/s41598-017-14829-4 PMC5665901

[38] M. Middlehurst, P. Schäfer, A. Bagnall, Bake off redux: A review and experimental evaluation of recent time series classification algorithms. Data Min. Knowl. Discov. 38, 1958–2031 (2024).

[39] N. Mohammadi Foumani, L. Miller, C. W. Tan, G. I. Webb, G. Forestier, M. Salehi, Deep learning for time series classification and extrinsic regression: A current survey. ACM Comput. Surv. 56, 1–45 (2024).

[40] H. I. Fawaz, B. Lucas, G. Forestier, C. Pelletier, D. F. Schmidt, J. Weber, G. I. Webb, L. Idoumghar, P.-A. Muller, F. Petitjean, InceptionTime: Finding AlexNet for time series classification. Data Min. Knowl. Discov. 34, 1936–1962 (2020).

