# Supplementary material for: One-stop strabismus digital diagnosis via AI-integrated skin-like and wearable “Eyelectronics”
Source: Sci Adv. 2026 Jan 28;12(5):eaeb7242. doi: 10.1126/sciadv.aeb7242 (PMC12851036; doi:10.1126/sciadv.aeb7242)
Supplement: Supplementary file 1 — Supplementary Text Figs. S1 to S17 Tables S1 to S6 Legends for movies S1 to S3 [file sciadv.aeb7242_sm.pdf]

Science Advances
AAAS

Supplementary Materials for

One-stop strabismus digital diagnosis via AI-integrated skin-like and wearable "Eyelectronics"

Yong Yang *et al.*

Corresponding author: Yonghong Jiao, yhjiao2001@aliyun.com; Yihao Chen, chenyihao92@tsinghua.edu.cn; Xue Feng, fengxue@tsinghua.edu.cn

Sci. Adv. **12**, eaeb7242 (2026)
DOI: 10.1126/sciadv.aeb7242

The PDF file includes:

Supplementary Text
Figs. S1 to S17
Tables S1 to S6
Legends for movies S1 to S3

Other Supplementary Material for this manuscript includes the following:

Movies S1 to S3

Supplementary Text

Note. S1. InceptionTime-Tiny Algorithm Process

(1) Perform channel and time differential processing on the original 3-channel strain signals $S \in \mathbb{R}^{T\times 3}$ to construct enhanced 10-channel input features:

$$X_{\text{aug}} = [S, \Delta S_{\text{channel}}, \Delta S_{\text{time}}] \in \mathbb{R}^{T\times 10}, \tag{1}$$

where $\Delta S_{\text{channel}}$ is the channel difference, ΔS_{time} is the first-order difference, generating a total of 10 channels.

(2) After receiving the 10-channel inputs mentioned above, the InceptionTime-Tiny model extracts temporal embedding vectors through multi-scale convolution blocks, bottleneck layers, and pooling,

$$z = f_{\text{Inception}}(X_{\text{aug}}; \theta) \in \mathbb{R}^{d}, \tag{2}$$

where $f_{\text{Inception}}$ represents the InceptionTime feature extraction network, θ represents the network parameters, and z is the embedding vector of length d.

Firstly, compress the channel dimension through a 1×1 convolution.

$$\boldsymbol{H}^{(0)} = \text{ReLU}\left(\boldsymbol{W}_{\text{bott}} * \boldsymbol{X}_{\text{aug}} + \boldsymbol{b}_{\text{bott}}\right), \tag{3}$$

where the size of the convolution kernel $\boldsymbol{W}_{\text{bott}} \in \mathbb{R}^{32\times 10\times 1}$ is 1 × 1, and the output dimension is $\boldsymbol{H}^{(0)} \in \mathbb{R}^{T\times 32}$. The 10-channel input is compressed into a 32-dimensional feature space to reduce the subsequent computational load.

Then, the Inception module is used for three branch parallel processing, that is, within a single Inception module, different scales of convolution kernels are used to independently process the input, and the results are concatenated and fused to form an architecture that captures short-term mutations (such as rapid scanning) and long-term trends (such as slow tracking) of eyelid strain signals, thereby improving sensitivity to small amplitude eye movements.

$$\boldsymbol{H}^{(1)} = \text{Concat}\left[\underbrace{\phi_{3\times 1}\left(\boldsymbol{H}^{(0)}\right)}_{\text{Branch 1}}, \underbrace{\phi_{7\times 1}\left(\boldsymbol{H}^{(0)}\right)}_{\text{Branch 2}}, \underbrace{\phi_{17\times 1}\left(\boldsymbol{H}^{(0)}\right)}_{\text{Branch 3}}\right], \tag{4}$$

where $\phi_{k\times 1}(\cdot)$ is the activation of one-dimensional convolution with kernel size k and ReLU. The final output is 32 channels per branch, and after splicing, it becomes 96 channels. Based on the biomechanical characteristics of eyelid strain signals, odd-numbered kernels are selected to ensure symmetrical filling, while different sizes of convolution kernels correspond to different time scales, because in the time domain, the size of the convolution kernels directly determines the size of the receptive field.

Then, by stacking multiple Inception modules, the shallow basic features are gradually combined into deep abstract features. By deepening the features, we can obtain,

$$\boldsymbol{H}^{(2)} = \text{Concat}\left[\phi_{3\times 1}\left(\boldsymbol{H}^{(1)}\right), \phi_{7\times 1}\left(\boldsymbol{H}^{(1)}\right), \phi_{17\times 1}\left(\boldsymbol{H}^{(1)}\right)\right]. \tag{5}$$

Then, perform adaptive feature pooling, which maps time-varying features to fixed-length vectors.

(3) Implement eye movement classification through a fully connected layer and output category probability vectors $p \in \mathbb{R}^4$,

$$\begin{aligned} p &= \operatorname{softmax}(W_c z + b_c), \\ \hat{y}_{\text{class}} &= \arg\max_i p_i, \end{aligned} \tag{6}$$

where $W_c \in \mathbb{R}^{4 \times d}$, $b_c \in \mathbb{R}^4$ are the classification weight and bias. $\hat{y}_{\text{class}}$ is the predicted direction category. Among them, the classification weights and biases are obtained through model training.

(4) For each direction classification result, input a multi-layer perceptron (MLP) network and output the angle coordinates,

$$(\hat{x}, \hat{y}) = f_{\text{MLP}}^{(\hat{y}_{\text{class}})}(z) \in \mathbb{R}^2 \tag{7}$$

where $f_{\text{MLP}}^{(\hat{y}_{\text{class}})}$ represents the exclusive MLP sub-models trained for each category.

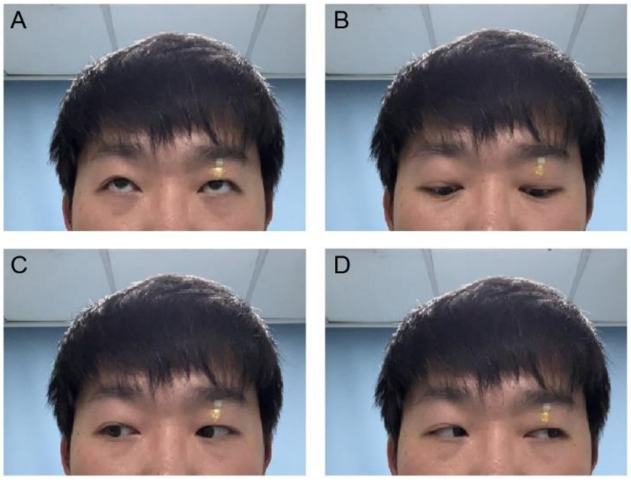

Fig. S1. Photographs showing the eye moving in four directions: (A) up, (B) down, (C) right, and (D) left, while wearing the Eyelectronics. It barely restricts eyelid deformation caused by eye movements.

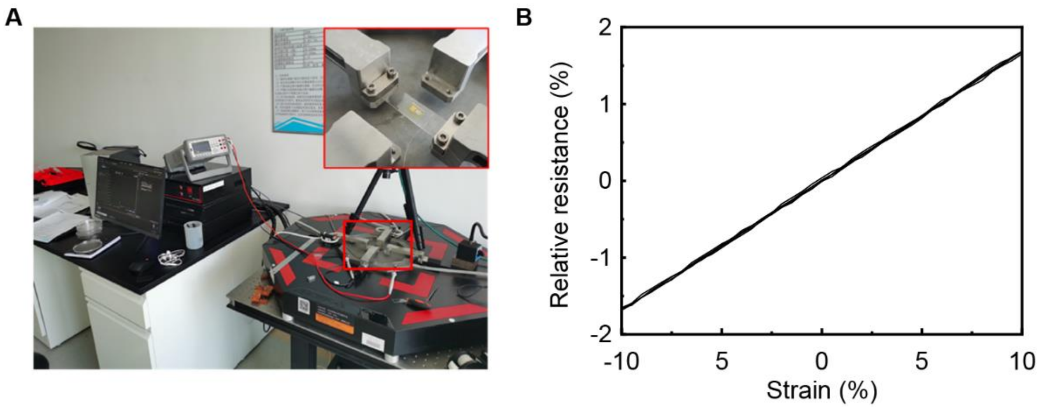

Fig. S2. Calibration of the strain sensing array. (**A**) Photograph of the calibration experiment with a tensile testing machine. Inset: The array is stretched and deformed. (**B**) Repeat performance of the array. The relationship between the relative resistance of the array and the applied strain is repeated for three cycles. The sensitivities of the array are 0.169, 0.169, 0.169, 0.169, 0.168, and 0.168, respectively.

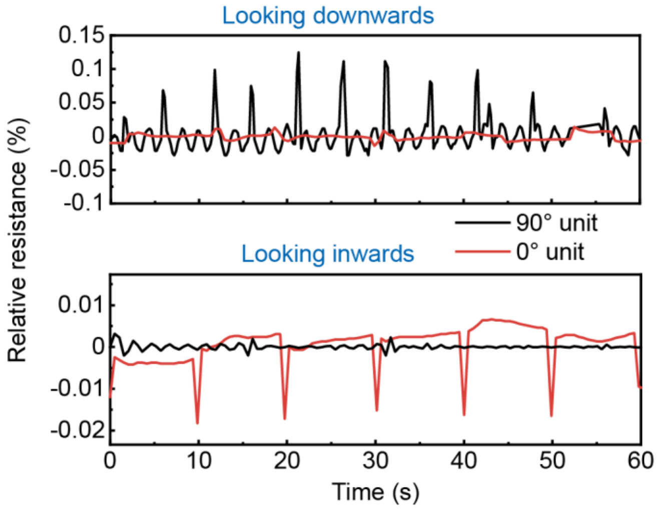

Fig. S3. Responses of 0° and 90° units to vertical and horizontal eye movements. 0° and 90° units are mainly responsible for sensing the horizontal and vertical deformation of the eyelid, respectively. Take the comparison of two unit signals when looking downwards and looking inwards as examples.

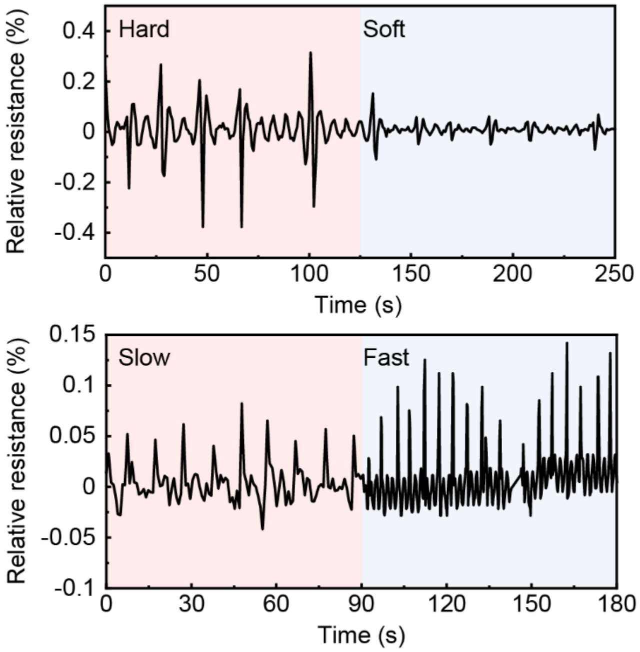

Fig. S4. 90° unit signals of different amplitudes and different frequencies. The amplitudes (approximately 5°–15°) and frequencies (approximately 0.1–1 Hz) were selected to mimic the typical range of quasi-static or slow saccadic eye movements observed under normal physiological conditions.

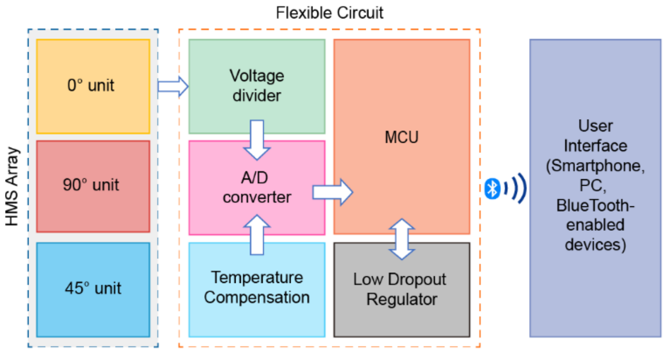

Fig. S5. Block diagram of the Eyelectronics, including the HMS array, the flexible circuit, and Bluetooth-enabled devices. The resistance values of three units are measured based on the voltage division method.

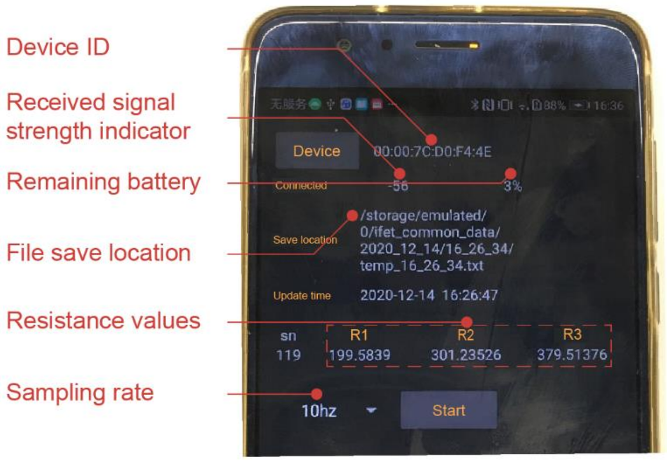

Fig. S6. Interface presentation of collecting resistance values of the HMS array with a smartphone via Bluetooth. It shows the device ID, received signal strength indicator, remaining battery, file save location, resistance values (R1, R2, and R3), and sampling rate. 5 Hz/ 10 Hz sample rates can be selected to accommodate wireless operation, ultrathin form factor, and thermal management, which are sufficient for static or quasi-static gaze assessments such as Hess screen tests. The orange words are translated from Chinese to English.

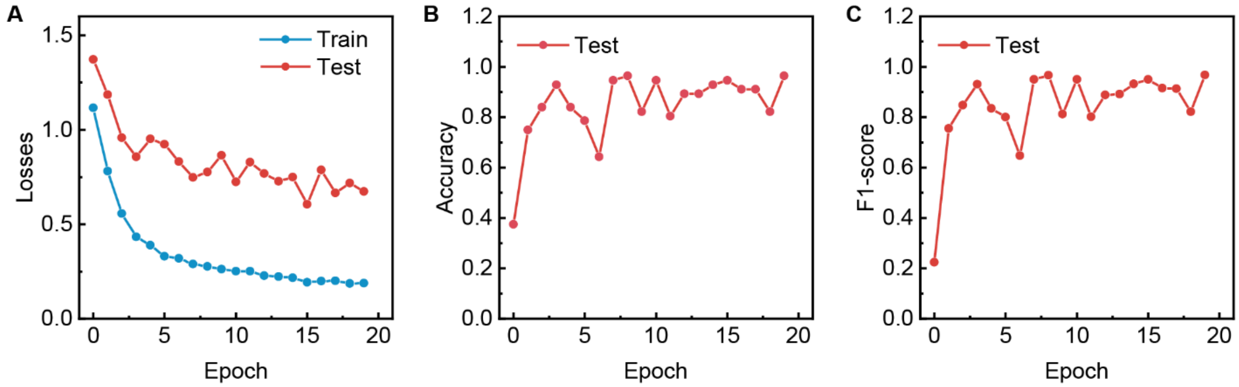

Fig. S7. Eye movement classification training process. (**A**) As the number of training iterations increases, the training loss continues to decrease, and the loss of the test set fluctuates and decreases. (**B**) The classification accuracy of the test set fluctuates and increases, reaching a maximum of 96.6%. (**C**) The F1-score of the test set fluctuates and increases, reaching a maximum of 96.7%.

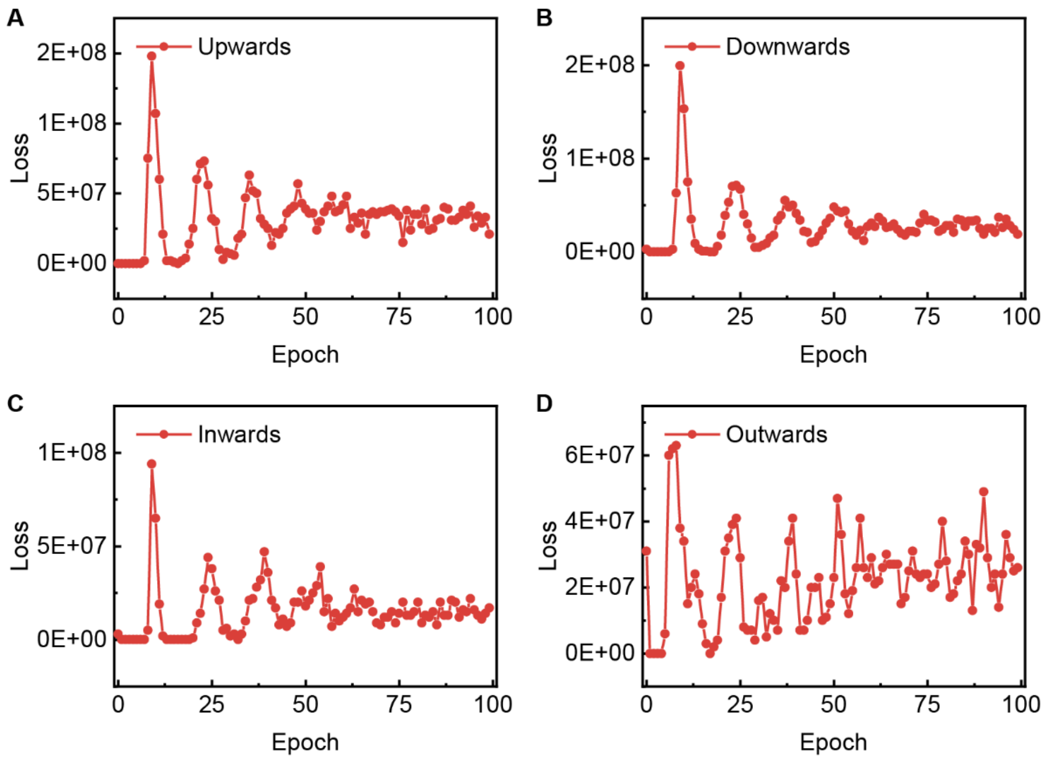

Fig. S8. Eye movement regression training process. (**A**), (**B**), (**C**), (**D**) As the number of training iterations increases, the loss of the test set for eye movement in the four directions fluctuates and decreases.

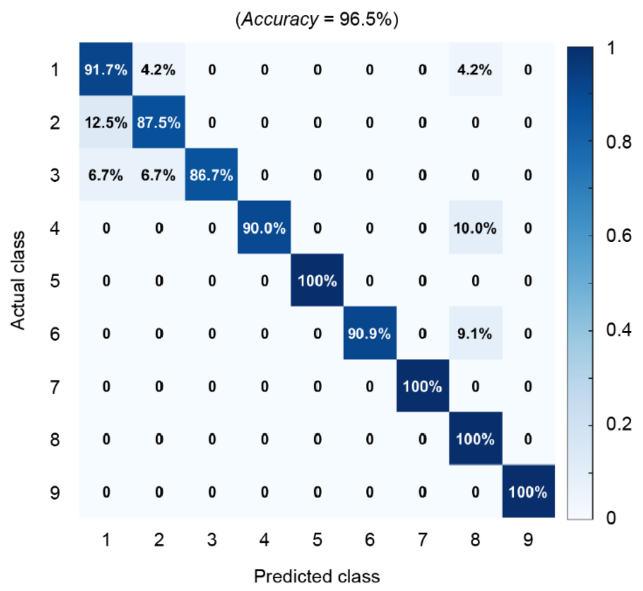

Fig. S9. Confusion matrix for the eight-direction classification of eye-movement and non-eye-movement signals, including blinking, happiness, and other facial expressions. Classes 1–9 stand for looking up, down, right, left, up-right, up-left, down-right, down-left, and interference containing blinking as well as facial expressions, respectively.

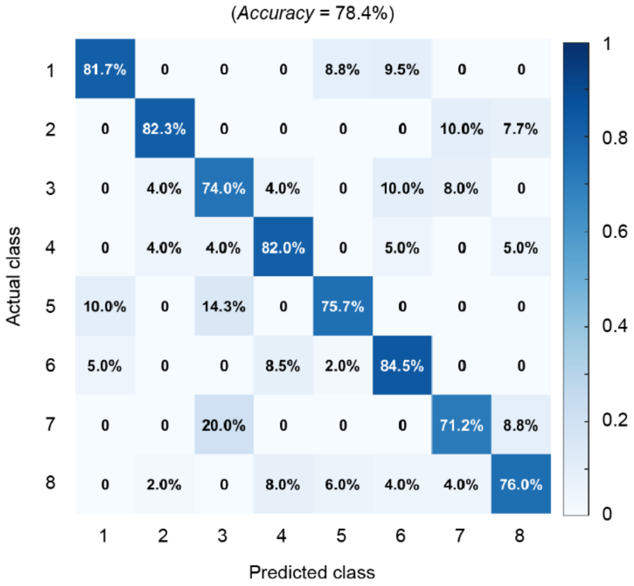

Fig. S10. Accuracy of eye movement classification based on the KNN-DTW method. Classes 1–8 stand for looking up, down, right, left, up-right, up-left, down-right, and down-left, respectively.

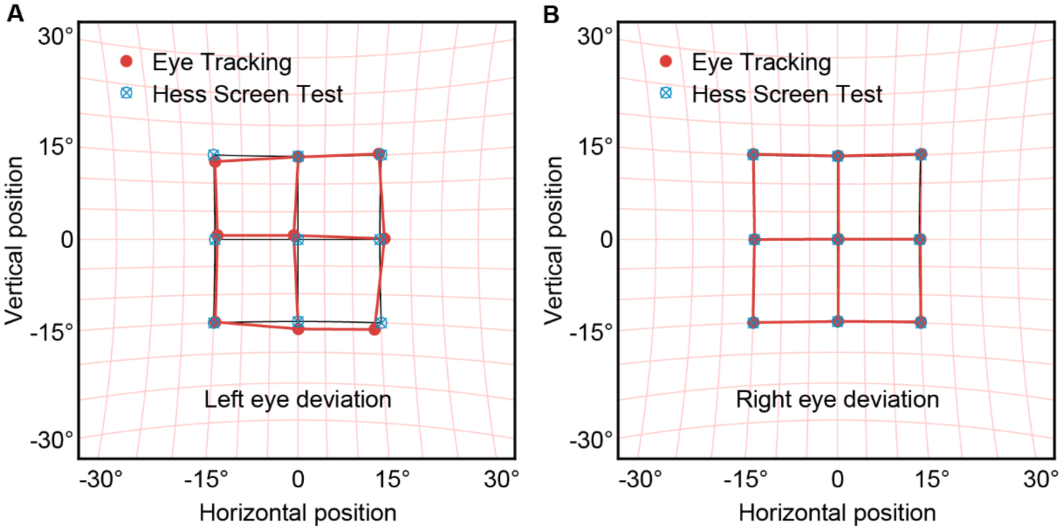

Fig. S11. Hess screen test for a normal subject. (**A**) Left eye deviation. (**B**) Right eye deviation. It can be seen that the traditional Hess screen test and the test based on the Eyelectronics have good consistency.

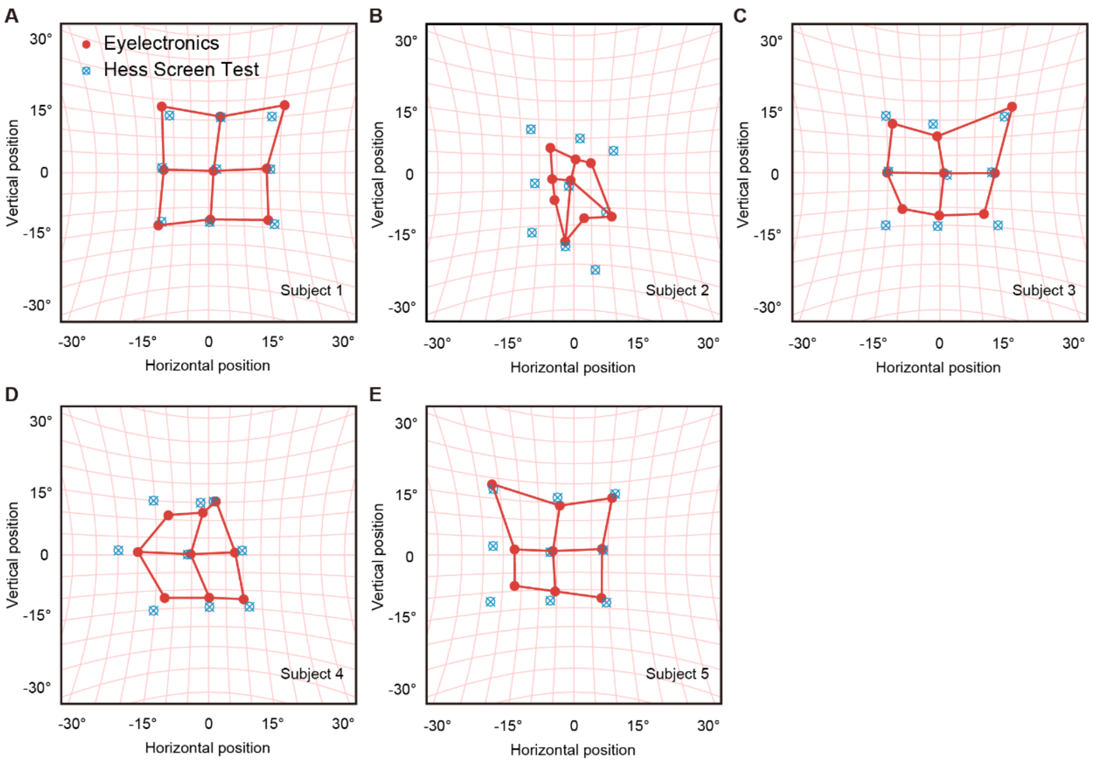

Fig. S12. Comparisons of the standard Hess screen test results and the test results using the Eyelectronics from five recruited patients.

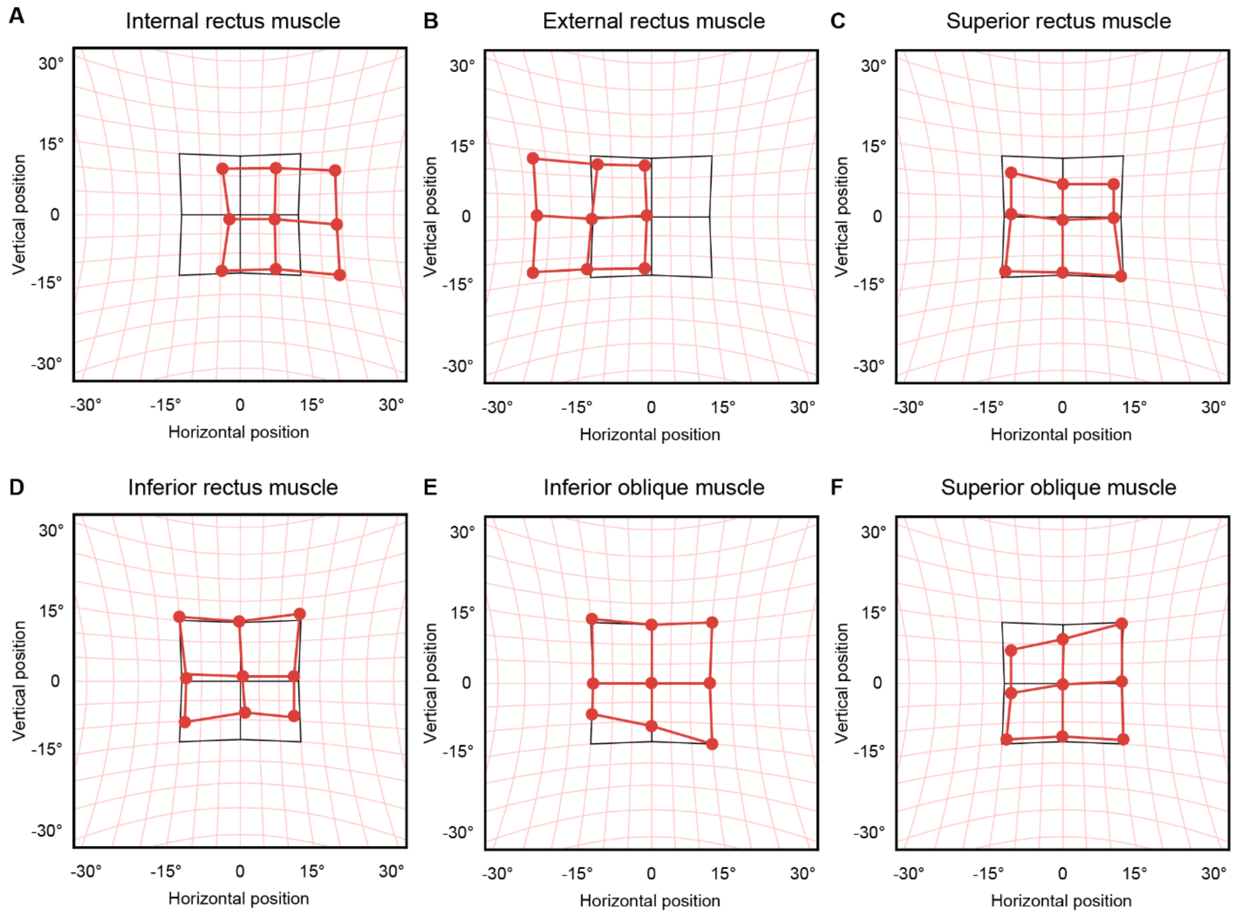

Fig. S13. Taking the right eye as an example, the typical Hess results of internal rectus muscle, external rectus muscle, superior rectus muscle, inferior rectus muscle, inferior oblique muscle, and superior oblique muscle paralysis. The result of the Hess screen for both eyes is asymmetric, which is known as paralytic strabismus. The smaller area of the Hess screen result is paralysis of the eye, and the outward deviation is paralysis of the medial rectus muscle. Therefore, threshold condition judgment based on Hess screen results and image visualization can be used to replace doctors in diagnosing paralyzed muscles.

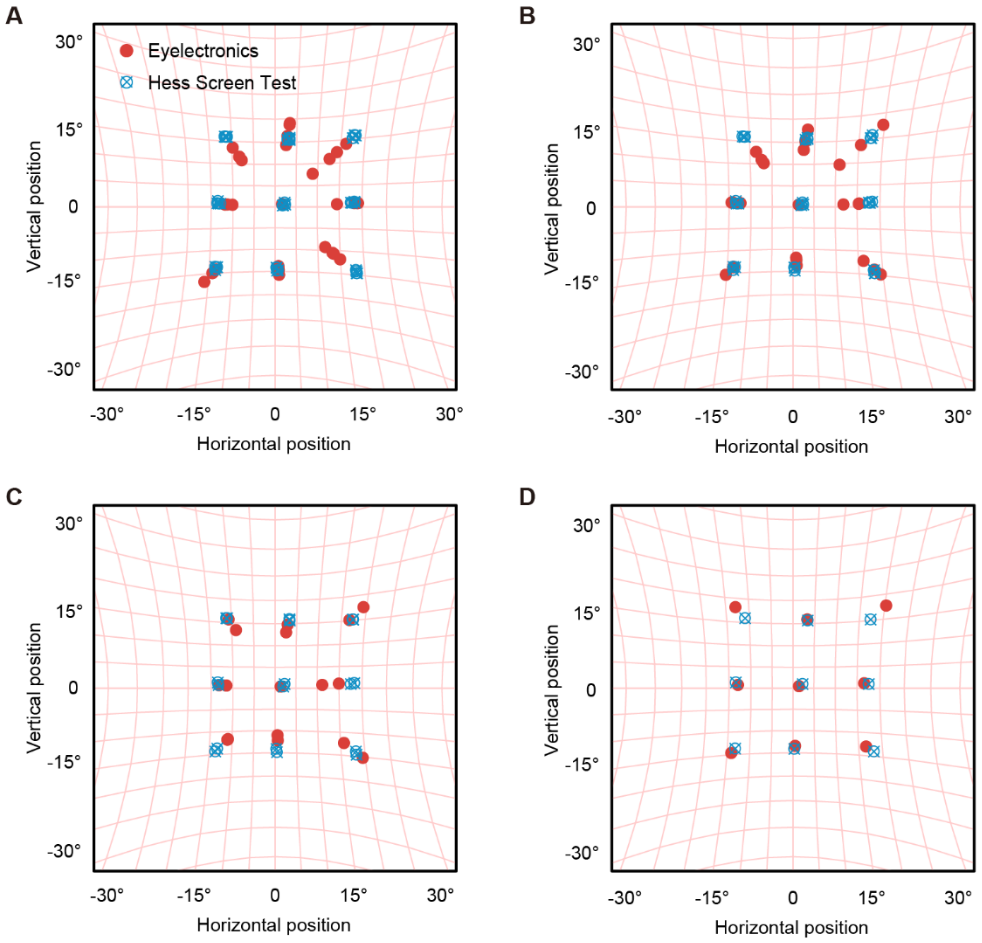

Fig. S14. Hess screen results obtained by fine-tuning the model with (A) 2 labeled samples, (B) 3 labeled samples, (C) 4 labeled samples, and (D) 5 labeled samples, showing decreasing MAE as the number of training samples increases.

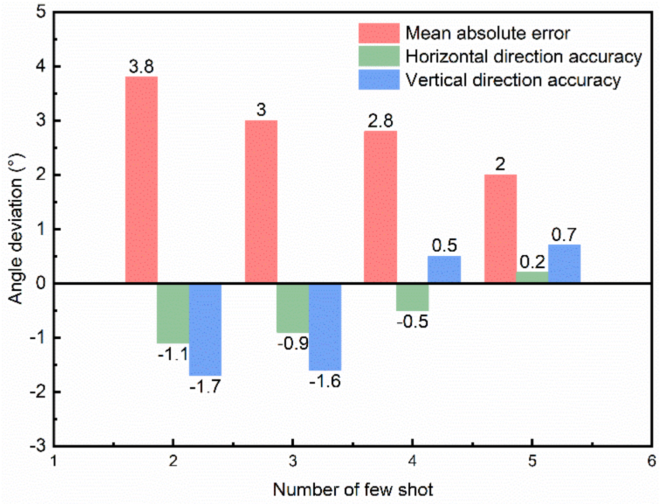

Fig. S15. MAE and directional accuracy gradually improve as the number of training samples increases.

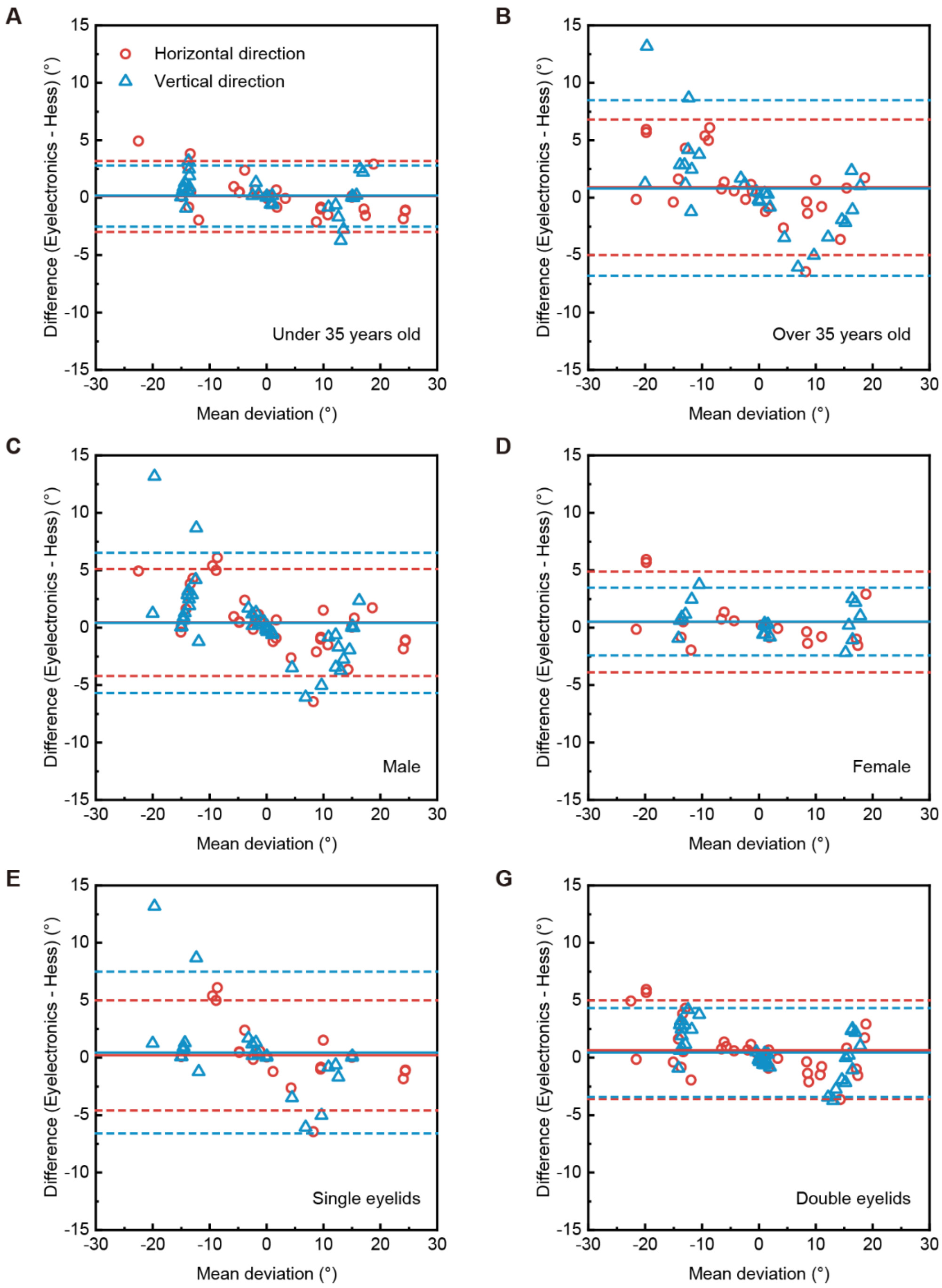

Fig. S16. Bland-Altman plot used to evaluate the agreement between two tests from stratified patients of age (<35 vs. ≥35 years), sex, and eyelid phenotype. Horizontal (solid red line) and vertical (solid blue line) mean differences are all close to zero. The 95% limits of agreement for vertical deviations (dashed blue line) and horizontal deviations (dashed red line) are not significantly different.

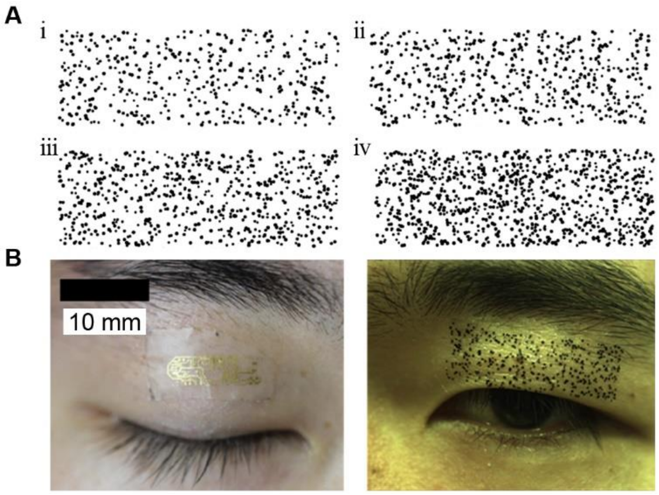

Fig. S17. Speckle tattoo designed for the digital image correlation method. (**A**) Schematic diagrams of sparser (i), sparse (ii), dense (iii), denser (iv) speckle patterns. (**B**) Comparison of a skin-like flexible electronic device and the speckle tattoo patch.

Table S1. Participant demographics and strabismus types

Subject ID	Age (years)	Sex	Eyelid phenotype	Strabismus type
S0	24	M	Single eyelid	Right hypotropic exotropia
S1	18	F	Single eyelid	Left hypertropic esotropia
S2	38	M	Single eyelid	Left hypotropic exotropia
S3	40	M	Double eyelid	Left esotropia
S4	32	M	Double eyelid	Right esotropia
S5	52	F	Single eyelid	Right hypertropic esotropia

Table S2. Results of Bias/LoA with confidence bands from stratified patients.

Stratification	Direction	Bias (95% CI: lower, upper) (°)	LoA lower bound (95% CI: lower, upper) (°)	LoA upper bound (95% CI: lower, upper) (°)
Under 35 years old	Horizontal	0.46 (-0.25, 1.16)	-4.16 (-5.38, -2.94)	5.07 (3.85, 6.29)
	Vertical	0.19 (-0.27, -0.65)	-2.45 (-3.24, -1.67)	2.84 (2.05, 3.63)
Over 35 years old	Horizontal	0.91 (-0.27, 2.10)	-4.96 (-7.01, -2.91)	6.79 (4.74, 8.84)
	Vertical	0.82 (-0.73, 2.36)	-6.84 (-9.51, -4.16)	8.48 (5.80, 11.15)
Male	Horizontal	0.46 (-0.25, 1.16)	-4.16 (-5.38, -2.94)	5.07 (3.85, 6.29)
	Vertical	0.43 (-0.50, 1.37)	-5.68 (-7.30, -4.07)	6.55 (4.93, 8.16)
Female	Horizontal	0.49 (-0.62, 1.61)	-3.91 (-5.85, -1.96)	4.89 (2.95, 6.84)
	Vertical	0.53 (-0.22, 1.28)	-2.41 (-3.71, -1.11)	3.47 (2.17, 4.77)
Single eyelid	Horizontal	0.20 (-0.77, 1.17)	-4.61 (-6.29, -2.93)	5.01 (3.33, 6.70)
	Vertical	0.46 (-0.95, 1.88)	-6.56 (-9.01, -4.10)	7.48 (5.03, 9.93)
Double eyelid	Horizontal	0.67 (-0.08, 1.41)	-3.65 (-4.93, -2.36)	4.98 (3.70, 6.26)
	Vertical	0.46 (-0.20, 1.12)	-3.37 (-4.50, -2.23)	4.28 (3.14, 5.42)

Table S3. MAE and direction accuracy with CIs per gaze position.

Gaze position	MAE (°) ± 95% CI	Horizontal direction accuracy (°) ± 95% CI	Vertical direction accuracy (°) ± 95% CI
Up-left	3.0 ± 1.9	1.3 ± 1.9	-1.3 ± 1.9
Up	2.3 ± 1.6	0 ± 0.6	-2.1 ± 1.7
Up-right	2.6 ± 1.8	-0.5 ± 2.3	-0.1 ± 1.5
Left	2.9 ± 1.8	2.6 ± 1.9	0.1 ± 0.6
Centre	1.0 ± 0.4	-0.1 ± 0.6	0.3 ± 0.5
Right	1.1 ± 0.6	-0.3 ± 0.9	-0.2 ± 0.4
Down-left	4.3 ± 2.8	2.7 ± 2.1	2.8 ± 2.4
Down	1.7 ± 0.8	0.1 ± 0.5	1.6 ± 0.8
Down-right	3.7 ± 3.4	-1.7 ± 0.9	3.0 ± 3.4

Table S4. MAE with CIs per gaze position from stratified patients.

Gaze position	MAE (°) ± 95% CI					
	**Under 35 years old**	**Over 35 years old**	**Male**	**Female**	**Single eyelid**	**Double eyelid**
Up-left	2.6 ± 2.1	3.6 ± 3.7	3.4 ± 2.5	2.0 ± 1.9	3.1 ± 4.3	3.0 ± 1.7
Up	1.2 ± 1.2	4.0 ± 2.3	2.8 ± 2.1	1.2 ± 2.0	2.5 ± 3.7	2.2 ± 1.4
Up-right	1.7 ± 1.6	3.9 ± 3.5	2.6 ± 2.5	2.6 ± 2.5	3.2 ± 4.2	2.2 ± 1.4
Left	2.2 ± 2.1	3.8 ± 3.2	2.7 ± 2.1	3.3 ± 4.9	2.7 ± 2.8	3.0 ± 2.7
Centre	0.8 ± 0.5	1.2 ± 0.6	1.0 ± 0.5	0.9 ± 0.2	1.0 ± 1.0	1.0 ± 0.1
Right	1.1 ± 0.8	1.1 ± 0.9	1.2 ± 0.7	0.7 ± 0.5	1.1 ± 1.0	1.1 ± 0.7
Down-left	1.6 ± 1.8	7.9 ± 2.8	4.4 ± 3.7	4.1 ± 5.7	3.8 ± 6.6	4.6 ± 2.5
Down	1.2 ± 1.0	2.3 ± 1.0	1.7 ± 0.9	1.7 ± 2.1	1.0 ± 0.8	2.2 ± 1.0
Down-right	1.4 ± 1.0	6.6 ± 6.9	4.4 ± 4.7	1.8 ± 0	5.0 ± 8.3	2.7 ± 1.3

Table S5. Horizontal direction accuracy with CIs per gaze position from stratified patients.

Gaze position	Horizontal direction accuracy (°) ± 95% CI					
	Under 35 years old	Over 35 years old	Male	Female	Single eyelid	Double eyelid
Up-left	0.6 ± 2.3	2.3 ± 3.2	2.3 ± 2.0	-1.1 ± 1.1	2.0 ± 3.3	0.8 ± 2.4
Up	-0.1 ± 0.6	0.2 ± 1.4	0.0 ± 0.9	0.2 ± 0.4	-0.7 ± 0.8	0.6 ± 0.5
Up-right	0.5 ± 1.9	-1.8 ± 4.7	-1.2 ± 2.8	1.1 ± 2.3	-2.7 ± 3.8	1.1 ± 1.5
Left	2.0 ± 2.1	3.4 ± 3.8	2.4 ± 2.2	3.1 ± 3.2	2.5 ± 2.7	2.7 ± 3.0
Centre	-0.2 ± 0.9	0.1 ± 1.0	-0.1 ± 0.8	-0.1 ± 1.0	-0.1 ± 0.9	0 ± 1.0
Right	-1.0 ± 0.9	0.7 ± 1.1	-0.2 ± 1.3	-0.7 ± 0.4	0.2 ± 1.5	-0.7 ± 1.2
Down-left	0.6 ± 1.5	5.4 ± 1.1	2.7 ± 2.2	2.5 ± 4.2	2.2 ± 3.8	3.0 ± 2.8
Down	-0.1 ± 0.5	0.5 ± 0.9	-0.1 ± 0.4	0.8 ± 0.7	-0.3 ± 0.6	0.5 ± 0.6
Down-right	-1.1 ± 0.7	-2.6 ± 1.3	-1.8 ± 1.2	-1.5 ± 0.1	-1.3 ± 1.5	-2.0 ± 1.1

Table S6. Vertical direction accuracy with CIs per gaze position from stratified patients.

Gaze position	Vertical direction accuracy (°) ± 95% CI					
	**Under 35 years old**	**Over 35 years old**	**Male**	**Female**	**Single eyelid**	**Double eyelid**
Up-left	-0.8 ± 2.5	-2.0 ± 3.4	-2.5 ± 1.7	1.6 ± 1.1	-2.2 ± 2.9	-0.6 ± 2.7
Up	-0.8 ± 1.4	-3.9 ± 2.2	-2.5 ± 2.1	-1.0 ± 2.3	-2.2 ± 3.8	-2.0 ± 1.5
Up-right	0.5 ± 1.4	-0.7 ± 3.3	-0.4 ± 1.8	0.7 ± 3.5	-1.4 ± 2.1	1.0 ± 1.7
Left	0.1 ± 0.9	0 ± 1.2	0.3 ± 0.7	-0.7 ± 0.2	0.9 ± 0.8	-0.6 ± 0.2
Centre	-0.1 ± 0.4	0.9 ± 0.8	0.5 ± 0.6	-0.1 ± 0.9	0.7 ± 1.0	0.1 ± 0.5
Right	0 ± 0.4	-0.4 ± 0.9	-0.3 ± 0.5	0.2 ± 0.1	-0.3 ± 0.9	-0.1 ± 0.4
Down-left	0.8 ± 1.7	5.5 ± 3.1	3.4 ± 3.0	1.4 ± 4.6	3.1 ± 5.4	2.5 ± 2.3
Down	1.1 ± 1.0	2.2 ± 1.0	1.6 ± 1.0	1.6 ± 1.8	0.9 ± 0.8	2.1 ± 1.0
Down-right	0.9 ± 0.8	5.7 ± 7.4	3.8 ± 4.7	1.0 ± 0.3	4.7 ± 8.3	1.7 ± 0.9

Movie S1. Eye tracking using the Eyelectronics.

Movie S2. Hess screen test process demonstration.

Movie S3. HMS array sensing strain and communicating with a smartphone.
